# Addressing the contribution of previously described genetic and epidemiological risk factors associated with increased prostate cancer risk and aggressive disease within men from South Africa

**DOI:** 10.1186/1471-2490-13-74

**Published:** 2013-12-29

**Authors:** Elizabeth A Tindall, MS Riana Bornman, Smit van Zyl, Alpheus M Segone, L Richard Monare, Philip A Venter, Vanessa M Hayes

**Affiliations:** 1 J. Craig Venter Institute, Genomic Medicine Group, San Diego, CA, USA; 2 (formerly) Children’s Cancer Institute Australia, Lowy Cancer Research Centre, University of New South Wales, Kensington, Sydney, Australia; 3 Department of Urology, University of Pretoria, Pretoria, South Africa; 4 Department of Urology, University of Limpopo, Medunsa Campus, Medunsa, South Africa; 5 Department of Medical Sciences, University of Limpopo, Turfloop Campus, Sovenga, South Africa; 6 Human Comparative and Prostate Cancer Genomics, The Garvan Institute of Medical Research, The Kinghorn Cancer Centre, Darlinghurst, Sydney, Australia

**Keywords:** Prostate cancer, African ancestry, Risk factors, Aggressive disease, Genetics, Epidemiology, Pilot analysis, Southern Africa

## Abstract

**Background:**

Although African ancestry represents a significant risk factor for prostate cancer, few studies have investigated the significance of prostate cancer and relevance of previously defined genetic and epidemiological prostate cancer risk factors within Africa. We recently established the Southern African Prostate Cancer Study (SAPCS), a resource for epidemiological and genetic analysis of prostate cancer risk and outcomes in Black men from South Africa. Biased towards highly aggressive prostate cancer disease, this is the first reported data analysis.

**Methods:**

The SAPCS is an ongoing population-based study of Black men with or without prostate cancer. Pilot analysis was performed for the first 837 participants, 522 cases and 315 controls. We investigate 46 pre-defined prostate cancer risk alleles and up to 24 epidemiological measures including demographic, lifestyle and environmental factors, for power to predict disease status and to drive on-going SAPCS recruitment, sampling procedures and research direction.

**Results:**

Preliminary results suggest that no previously defined risk alleles significantly predict prostate cancer occurrence within the SAPCS. Furthermore, genetic risk profiles did not enhance the predictive power of prostate specific antigen (PSA) testing. Our study supports several lifestyle/environmental factors contributing to prostate cancer risk including a family history of cancer, diabetes, current sexual activity and erectile dysfunction, balding pattern, frequent aspirin usage and high PSA levels.

**Conclusions:**

Despite a clear increased prostate cancer risk associated with an African ancestry, experimental data is lacking within Africa. This pilot study is therefore a significant contribution to the field. While genetic risk factors (largely European-defined) show no evidence for disease prediction in the SAPCS, several epidemiological factors were associated with prostate cancer status. We call for improved study power by building on the SAPCS resource, further validation of associated factors in independent African-based resources, and genome-wide approaches to define African-specific risk alleles.

## Background

To-date, there is little evidence implicating any carcinogens or modifiable risk factors to prostate cancer development and subsequent progression. Furthermore, there is currently no known cure for metastatic disease. Henceforth, the greatest hope of minimizing the impact of prostate cancer is early detection and intervention. A major consequence of this approach however, is the potential for over-diagnosis and over-treatment of individuals whose disease may never eventuate to mortality. Regular screening in developed nations for the most commonly used marker of prostate cancer to-date, prostate specific antigen (PSA) levels has become somewhat controversial. Risk stratification studies within Europe and the USA [1-3] have prompted recommendations from both the American Urological Association and American Cancer Society to only screen men with a life-expectancy exceeding 10 years. Furthermore, the U.S. Preventive Services Task Force have removed their recommendation for regular PSA screening of asymptomatic men, regardless of age [4]. The heterogeneous nature of prostate cancer and the potential of indirect influences affecting PSA levels, has prompted investigations into alternate prognostic marker development, particularly the early detection of aggressive disease. Alternative markers such as prostate cancer antigen 3 (PCA3) [5] and several genetic risk profiles [6,7] have been proposed to compliment PSA screening methods, but these methods are still being fine-tuned for clinical application.

**Figure 1:**
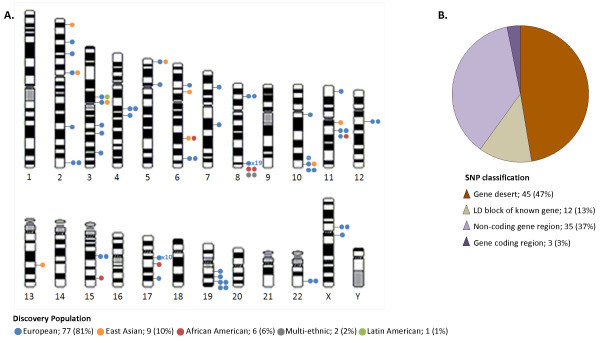
**Current content of prostate cancer risk alleles. (A)** Chromosomal distribution and discovery population of published prostate cancer risk alleles achieving genome-wide significance (P-value < 10^-6^). Each dot represents one single nucleotide polymorphism (SNP), except where a numerical value indicates the number of SNPs that dot represents (applicable to regions 8q24 and 17q12). Dots are color coded to represent the discovery population of each SNP. **(B)** Classification of each SNP represented in Figure **(A)**, relative to known, characterized genes.

In addition to increasing age and a familial history of the disease, an African ancestry is one of the few known risk factors of prostate cancer [8-14]. Despite this fact, prostate cancer genome-wide association studies (GWAS) have to-date been predominantly European biased. As of October 2012, a total of 94 single nucleotide polymorphisms (SNPs) across 41 chromosomal loci were reported to achieve a genome-wide significance level, which we conservatively define in this report as a p-value < 10^-6^ in order to maximize capture of potential loci, across 18 GWAS (Figure 1 and Additional file 1: Table S1). Of the 18 GWAS, a total of 15 (83%) were performed within a European (including one Latin American [15]) study population, two on a Japanese cohort [16,17] and a single GWAS has exclusively targeted African American’s [18].

European study bias is not only evident by the disproportionate focus of GWAS discoveries in European men (77/95; 81%), but also in the current content of popular genome-wide arrays, which best represent individuals of European (53-93% genomic coverage dependent on array size) and East Asian (55-92% coverage) ancestry and to a lesser extent Yoruba of West Africa (31-73% coverage) [19]. The more recent release of 2.5 and 4–5 million marker arrays has improved rare variant content but not shown a marked improvement in Yoruba genomic coverage (approximately 71% coverage).

Due to the predicted complexity of prostate cancer, epidemiological factors including occupational hazards [20,21], dietary factors and other health related issues (reviewed in [22]), as well as several hormone influenced conditions such as male pattern baldness [23], have also been proposed to play a role in prostate cancer pathogenesis. However, investigations have similarly been largely focused on European populations.

The goal of the Southern African Prostate Cancer Study (SAPCS) is to provide a unique resource, un-biased by non-African admixture or by routine PSA screening practices of Western societies, to investigate the inherited genetic contribution as well as epidemiological (including environmental and lifestyle factors) influences on global disparities in prostate cancer, particularly associated with men of African descent. The power of this study is not only in the comprehensive analysis of known risk factors in an as yet un-investigated, potentially high risk population (pertaining to a pure African ancestral contribution), but also in the inclusion of study participants presenting overwhelmingly with an aggressive disease phenotype. This is the first analysis of genetic and epidemiological data within the SAPCS and the first study of its kind for Black South African men.

## Methods

### Study design and inclusion

All men contributing to the SAPCS in this report are defined as Black South African’s, specifically they all self-defined as Southern Bantu speakers, a term used to group the majority of Bantu languages spoken in South Africa, Botswana and Mozambique. Although we recognize that it is grammatically correct to use prefixes when referring to a Bantu people or language, for simplicity, in this report, we will refer to participating Southern Bantu groups by the English derived ethno-linguistic identifiers. The population contributions and substructure of the SAPCS participants, the latter based on genome-wide genetic analysis, is defined elsewhere [Tindall et al., submitted]. We recognize a unique within Africa Southern Bantu population clustering.

**Figure 2:**
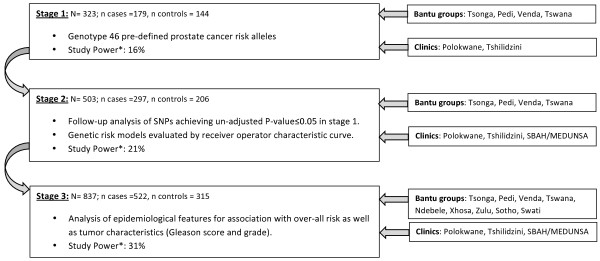
**Stages 1 to 3 of SAPCS data analysis.** Depicting study size, study inclusion (Bantu population groups and participating urological clinics), data analysis and study power at first three stages of SAPCS collection. *Study Power represents power to detect an OR = 1.4, given a probability of exposure in controls = 0.1 in single hypothesis testing.

The SAPCS is an ongoing longitudinal collection initiated in 2008 with approval by research ethics and institutional review boards at the Limpopo Provincial Government and University of Limpopo, South Africa (#32/2008 and MREC/H/28/2009), University of Pretoria, South Africa (#43/2010), J. Craig Venter Institute, U.S.A. (#2010-129) and the University of New South Wales, Australia (#H00/088). As recruitment is ongoing, this manuscript presents research performed at the first three stages of collection as outlined in Figure 2. Participants have to-date been sourced from urological clinics within South Africa including, Polokwane Hospital, Tshilidzini Hospital, Pretoria’s Steve Biko Academic Hospital (SBAH) and the Medical University of South Africa (MEDUNSA), representing both rural and urban based clinics respectively. As routine PSA testing is not common practice in these regions, study participants presented at the clinic largely as a result of urological complaints. Subjects were reviewed by local urologists, PSA testing performed, and prostate cancer status defined histopathologically by Gleason score and tumor grade (well, moderate and poor differentiation). Control samples were those diagnosed with either benign prostatic hyperplasia (BPH) and/or absence of any clinically definable prostate cancer. A clinical and demographic summation of patients and controls within the SAPCS has been presented elsewhere, including the evolution and cultural limitations of establishing a prostate cancer study within the constraints of Africa [Tindall et al., submitted]. As of April 2010 (stage 1) there were 323 participants, 179 cases and 144 controls, by October 2011 (stage 2) the study totaled 503 subjects, 297 cases and 206 controls, and was largely restricted to four ethnolinguistic populations (the Pedi, Tsonga, Venda and Tswana) and by the time of data analysis (stage 3) the study had been expanded to include all peoples defined as Southern Bantu and included 837 participants, 522 cases and 315 controls. Pilot analysis allows for assessing recruitment validity as well as providing statistical trends to drive future research focus.

### Study material and data collection

From each participant, a minimum of 800 ng DNA was extracted from whole blood using Qiagen QIAmp DNA mini kits. Participants were also requested to complete a questionnaire during a face-to-face interview with an attending professional urologist or nurse. A summary of the questionnaire will be made available on the study webpage at http://www.SAPCS.Webs.com.

### SNP selection

All SNPs reported to reach genome-wide significance, defined here as a P-value < 1x10^-6^, and published prior to April 2010 were included in our preliminary stage 1 analysis (44 SNPs; Figure 1 and Additional file 1: Table S1). Two additional SNPs included, Bd11934905 (8q24 region 2), which did not achieve genome-wide significance (P-value = 1.5x10^-4^) in the discovery target study, but was unique in the African American population [24] and rs7210100 (17q21), which achieved genome-wide significance in the first published African American prostate cancer GWAS (P-value = 3.4x10^-13^), but was published after the stage 1 publication deadline [18].

### Genotyping

Stage 1 genotyping was performed using a custom designed Illumina Universal Array Matrix (UAM). Quality control (QC) inclusion values were a GenTrain score > 0.5 and a call rate > 0.9 (SNP and sample). The rs7210100 variant was genotyped via direct Sanger sequencing. SNPs regarded as significant (P-value ≤ 0.05) prior to correction for multiple testing, were genotyped on additional samples (stage 2) also via direct Sanger sequencing. Primer information and amplification conditions are available upon request.

### Statistical analysis

For disease association testing we report Fischer’s exact P-value, per allele odds ratios (OR) and 95% confidence intervals (CI) estimated by logistic regression using SVS version 7. Q-values, generated using a ‘smoothing’ method suggested by Storey and Tibshirani [25] provide significance levels adjusted for multiple testing by correcting for false discovery rate are generated using the QVALUE software package on R. Genotype-phenotype interactions were assessed for variants genotyped in stage 2 analysis using logistic regression models. Phenotypic characteristics applicable to both cases and controls, including PSA levels and family history of prostate cancer and all cancers combined were assessed in both cases and controls combined. Characteristics applicable only to prostate cancer cases, including age at presentation, Gleason score and tumor grade, were assessed in cases samples only. The effect of risk alleles on PSA levels, were further controlled for age as a potential confounding factor to this phenotype. Q-values for this subset of variants (thus a reduced number of hypothesis tested) were generated using the Benjamini and Hochberg method [26]. Power calculations were performed using PS Power and Sample Size Calculation version 3.0.43.

Genetic risk scores (GRS) were calculated using three alternative risk models (Addition file 2: Figure S1) for 3, 6 and 38 SNPs each. The 38 SNPs represent all stage 1 genotyped SNPs that passed QC, 6 SNPs represent all stage 2 genotyped SNPs and 3 SNPs represent SNPs that achieved an un-corrected P-value < 0.05 in stage 2 analysis. Models 1 and 2 are derived as a count of risk alleles. Model 1, count GRS (cGRS), reflects the number of risk alleles only (cGRS = ∑risk alleles), and Model 2, weighted GRS (wGRS), is weighted according to the population specific allelic ORs (wGRS = LN OR∑risk alleles). Model 3 calculates the risk of each genotype combination (OR^n risk alleles) relative to the average population risk (∑population frequency x genotypic risk) in this case, SAPCS control population. Receiver Operating Characteristic (ROC) curves were generated to evaluate the performance of each genetic risk model and area under the curve (AUC) calculated to estimate the predictive power of each model [27]. We further assess the impact of combining PSA measures and the most accurate GRS presented here, wGRS for 38 SNPs.

Associations between prostate cancer status and various epidemiological parameters were first assessed by crude analysis using Pearson’s Chi-squared test with Yates’ continuity correction for dichotomous variables or Welch two sample T-test comparing means in continuous variables. Multiple logistic regression was also performed on each variable accounting for confounders including age, family history of prostate cancer and self-reported population group. Where a parameter contains >2 response variables, the condition most equally represented in both cases and controls was used as the reference. Furthermore, we performed multinomial logistic regression analysis to assess possible associations between various clinical features including Gleason score and tumor grade.

## Results

### Stage 1: Genotype analysis

We recently described the Southern Bantu population groups included in this study as forming a single genetic cluster distinct from other African populations including both Eastern and Western Bantu-derived populations, supporting the combined analysis of Southern Bantu men for genetic association studies [Tindall et al., submitted]. We genotyped a total of 46 SNPs for stage 1 analysis (183 cases and 146 controls). Four case samples (two Pedi and one each Tsonga and Venda) and two control samples (both Tsonga) failed to achieve a call rate > 0.9 so were excluded (leaving a total of 179 cases and 144 controls). Eight variants failed QC parameters, including rs7000448, rs10993994, rs8102476, rs12500426, rs1016343 (call rate < 0.9), rs620861 (GenTrain score < 0.5) and two variants, rs12621278 and rs6983267, presented with a minor allele frequency (MAF) = 0. Of the remaining 38 SNPs, six achieved a P-value ≤ 0.05 prior to correcting for multiple testing; rs10090154-T (OR = 0.55 95%CI 0.36-0.83; P-value = 0.0044), rs6983561-C (OR = 1.54 95% CI 1.12-2.13; P-value = 0.0094), rs13254738-C (OR = 0.70 95% CI 0.50-0.96; P = 0.0306), rs1859962-G (OR = 1.50 95% CI 1.03-2.20; P = 0.0379), rs1465618-A (OR = 1.80 95% CI 1.02-3.16; P = 0.0423), and rs4242382-A (OR = 0.07 95% CI 0.50-1.00; P = 0.0503) (Table 1 and Additional file 1: Table S1). After correcting for false discovery rate, Q-values were all >0.05. Four of the six SNPs with un-adjusted P-values ≤ 0.05 lie within the 8q24 region (rs10090154, rs6983561, rs13254738, and rs4242382), one within 17q24 (rs1859962) and one at 2p21 (rs1465618). Of note is that the T allele of rs10090154 showed a protective effect in our study (OR = 0.55, 95% CI 0.36-0.83), in contrast to previous reports including the discovery European-based GWAS, which suggests the T allele is associated with increased prostate cancer risk (OR = 1.67) [28].

**Table 1:** Stage 1 and 2 genotype results for SNPs achieving uncorrected P-value ≤ 0.05 in stage 1 analysis

		**Stage 1 (179 cases, 144 controls)**				**Stage 2 (297 cases, 206 controls)**			
**Marker (risk allele)**	**Location**	**MAF cases**	**MAF controls**	**OR (95% CI)**	**P-value**	**MAF cases**	**MAF controls**	**OR (95% CI)**	**P-value**
rs6983561 (C)	8q24 region 2	0.478	0.372	**1.54 (1.12-2.13)**	**0.0094**	0.505	0.407	**1.45 (1.15-1.92)**	**0.0024**
rs1859962 (G)	17q24	0.270	0.197	**1.50 (1.03-2.20)**	**0.0379**	0.260	0.199	**1.42 (1.05-1.93)**	**0.0276**
rs13254738 (C)	8q24 region 2	0.331	0.416	**0.70 (0.50-0.96)**	**0.0306**	0.323	0.389	**0.75 (0.58-0.98)**	**0.0363**
rs10090154 (T)	8q24 region 1	0.138	0.226	**0.55 (0.36-0.83)**	**0.0044**	0.167	0.212	0.74 (0.54-1.03)	0.0815
rs4242382 (A)	8q24 region 1	0.253	0.325	0.07 (0.50-1.00)	0.0503	0.269	0.299	0.87 (0.66-1.44)	0.3189
rs1465618 (A)	2p21	0.118	0.069	**1.80 (1.02-3.16)**	**0.0423**	0.098	0.081	1.23 (0.79-1.92)	0.4336

Stage 1 and stage 2 association analysis with over-all prostate cancer risk was achieved by comparing minor allele frequencies (MAF) in cases versus controls. Allelic odds ratios (OR) and 95% confidence intervals (CI) were estimated using logistic regression models. We report un-corrected P-values derived using Fischer’s exact test. Association results for alleles achieving un-corrected P-value < 0.05 are represented in bold type.

### Stage 2: Follow-up genotype and genetic risk score analysis

Six variants achieving an un-corrected P-value ≤ 0.05 in Stage 1, were genotyped on additional stage 2 samples, for a combined study size of 503 (297 cases and 206 controls). Only three SNPs achieved un-adjusted P-values < 0.05 in stage 2 analysis; rs6983561 (OR = 1.45 95% CI 1.15-1.92; P = 0.0024), rs1859962 (OR = 1.42 95% CI 1.05-1.93; P = 0.0276), and rs13254738 (OR = 0.75 95% CI 0.58-0.98; P = 0.0363; Table 1 ‘Stage 2’).

Interaction between the six variants genotyped in stage 2 analysis and various clinical and demographic characteristics reveals an unadjusted significant association between the C-allele of rs6983561 and serum PSA levels above 20 μg/L (OR = 1.35, 95% CI 1.04-1.74; P-value = 0.0224). Cases defined as having a poor tumor grade were significantly associated with rs6983561 (OR = 1.73, 95% CI 1.03-2.92; P = 0.0391) and rs1859962 (OR = 1.84, 95% CI 1.05-3.22; P = 0.0351; Table 2). Additional interactions between genotype and demographic/clinical features addressed for these SNPs include age at prostate cancer presentation, Gleason score, family history of prostate cancer and all cancers combined.

**Table 2:** Genotype-phenotype association analysis for variants genotyped in stage 2 analysis

**Characteristic**	**PSA**^**† **^**(n = 462)**			**Family PCa**^**† **^**(n = 458)**			**Family Ca**^**† **^**(n = 451)**			**Age of PCa presentation**^**¥ **^**(n = 265)**			**Gleason score**^**¥ **^**(n = 180)**			**Grade**^**¥ **^**(n = 147)**		
**Marker (risk allele)**	**OR (95% CI)**^‡*^	**P**^**§**^	**Q**^**¶**^	**OR (95% CI)**^**‡**^	**P**^**§**^	**Q**^**¶**^	**OR (95% CI)**^**‡**^	**P**^§^	**Q**^**¶**^	**OR (95% CI)**^**‡**^	**P**^**§**^	**Q**^**¶**^	**OR (95% CI)**^**‡**^	**P**^**§**^	**Q**^**¶**^	**OR (95% CI)**^**‡**^	**P**^**§**^	**Q**^**¶**^
rs6983561 (C)	**1.35 (1.04-1.74)**	**0.02**	0.40	1.40 (0.86-2.27)	0.18	0.60	0.91 (0.51-1.62)	0.75	0.84	0.81 (0.57-1.15)	0.24	0.68	1.11 (0.72-1.71)	0.63	0.81	**1.73 (1.03-2.92)**	**0.04**	0.35
rs1859962 (G)	1.28 (0.96-1.71)	0.09	0.60	1.00 (0.61-1.65)	0.99	0.99	0.58 (0.29-1.16)	0.12	0.60	0.95 (0.64-1.42)	0.82	0.86	1.22 (0.76-1.95)	0.42	0.76	**1.84 (1.05-3.22)**	**0.03**	0.35
rs13254738 (C)	1.15 (0.87-1.52)	0.33	0.60	1.04 (0.61-1.79)	0.88	0.93	1.12 (0.67-1.89)	0.66	0.79	1.06 (0.73-1.54)	0.75	0.86	0.72 (0.46-1.13)	0.15	0.68	1.16 (0.67-1.99)	0.60	0.81
rs10090154 (T)	0.89 (0.64-1.24)	0.50	0.75	0.70 (0.34-1.43)	0.33	0.60	1.44 (0.74-2.79)	0.29	0.60	1.03 (0.64-1.65)	0.91	0.91	0.79 (0.43-1.44)	0.43	0.76	1.28 (0.66-2.47)	0.47	0.76
rs4242382 (A)	0.81 (0.61-1.09)	0.17	0.60	0.87 (0.50-1.50)	0.62	0.79	1.20 (0.66-2.18)	0.54	0.75	1.06 (0.72-1.57)	0.78	0.86	1.21 (0.74-1.96)	0.45	0.76	1.46 (0.83-2.56)	0.19	0.68
rs1465618 (A)	1.29 (0.80-2.08)	0.29	0.60	1.46 (0.68-3.16)	0.33	0.60	1.43 (0.58-3.47)	0.44	0.71	0.71 (0.39-1.29)	0.26	0.68	1.54 (0.80-2.95)	0.20	0.68	1.30 (0.56-3.01)	0.54	0.81

^**†**^Genotype-phenotype association analysis for PSA **(<**20 μg/L = 0, ≥20 μg/L = 1), Family PCa (defined as first degree relatives affected by prostate cancer; no = 0, yes = 1) and Family Ca (defined as first and second degree relatives affected with any cancer; no = 0, yes = 1) was performed using all available samples (cases and controls).

^**¥**^Genotype-phenotype association analysis for Age of prostate cancer presentation (<70 yrs = 1, ≥70 yrs = 0), Gleason score (≤7 = 0, >7 = 1) and tumor Grade (well/moderate = 0, poor = 1) was performed on prostate cancer cases only.

^‡^OR (odds ratios) and 95% CI (confidence intervals) were estimated using unconditional logistic regression models.

*PSA estimates were additionally controlled for Age.

^§^Un-corrected P-values derived using logistic regression analysis.

^¶^Benjamini and Hochberg’s method to correct for false discovery rate [26] was used to generate Q-values.

**Figure 3:**
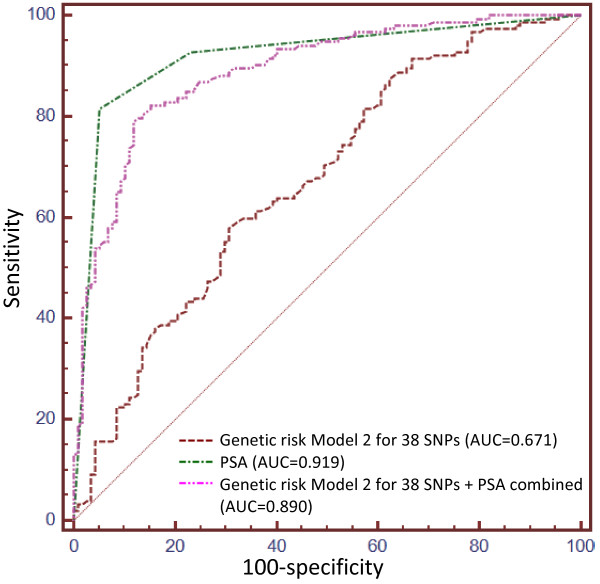
**Receiver operating characteristic (ROC) curves for genetic risk model 2 compared to PSA levels alone and combined with genetic risk model.** Genetic risk model 2 for 38 SNPs results in an AUC of 0.671. PSA levels more accurately predict prostate cancer occurrence in the SAPCS study population with an AUC of 0.919 (difference between areas = 0.248, 95% CI 0.176-0.319, P < 0.0001). The predictive power of PSA was not improved when combined with genetic risk model 2 for 38 SNPs (AUC = 0.890, difference between areas = 0.0292, 95% CI -0.003-0.062, P = 0.0784).

Three genetic risk models were applied to measure the combined genetic risk of the various SNPs tested. For each model the AUC, which measures the performance of each model, improved as the number of SNPs increased (Additional file 2: Figure S1A-C). This difference was only significant for models 2 and 3 when comparing each the 6 SNP and 38 SNP combinations to 3 SNPs. For Model 2 the difference between AUC relative to 3 SNPs is 0.035 (95% CI 0.005-0.065, P-value = 0.0208) and 0.065 (95% CI 0.015-0.116, P-value = 0.0117) for 6 and 38 SNPs respectively. For Model 3, the difference between AUC relative to 3 SNPs is 0.047 (95% CI 0.005-0.088, P-value = 0.0282) and 0.093 (95% CI 0.027-0.159, P-value = 0.0061) for 6 and 38 SNPs respectively. Model 2, which applies wGRS calculations, achieved a greater AUC compared to Models 1 and 3 for all *n* SNP combinations, although the difference was not statistically significant (Additional file 2: Figure S1D-F). Genetic risk Model 2 for 38 SNPs resulted in the largest AUC of all models tested (AUC = 0.671) and was therefore evaluated against the most common marker of prostate cancer to date, serum PSA levels, to predict prostate cancer in this study population (Figure 3). We determine PSA levels to be a more accurate predictor of prostate cancer than genetic risk Model 2 for 38 SNPs, with an AUC of 0.919 (difference between areas = 0.248, 95% CI 0.176-0.319, P-value < 0.0001). Combining the genetic risk model presented here with serum PSA levels did not improve the predictive capability of serum PSA alone and actually showed a slight (though non-significant) reduction in AUC to 0.890 (difference between areas = 0.0292, 95% CI -0.003-0.062, P-value = 0.0784).

### Stage 3: Epidemiological analysis

**Figure 4:**
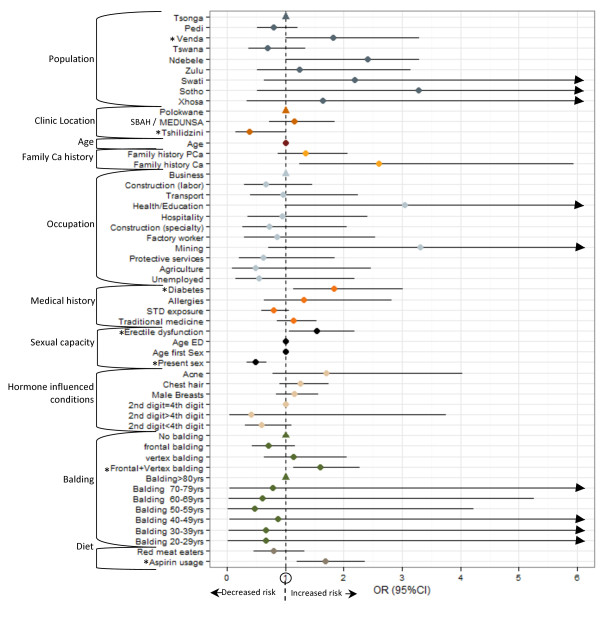
**Multiple logistic regression analysis for association between multiple variables and prostate cancer risk.** For each variable, odds ratios (ORs) are represented by dots and Confidence intervals (CI) are represented by horizontal lines extending from dots. Dots are color coded according to variable groupings indicated on left hand side of Y-axis. Reference groups for variables with multiple outcomes are represented by a single triangle (no CI) positioned along the Y-axis at X = 1. The X-axis limit was set at a value of 6.0. CI exceeding this limit are indicated by arrows. PSA levels are not represented on this plot due to values exceeding this limit. *indicates variable is associated with over-all prostate cancer risk at a significance level <0.05.

Crude analysis of epidemiological data revealed the overall distribution of several variables differed significantly between cases and controls; including population group (P-value = 0.0046), a family history of cancer (P-value = 0.0434), previous occupation (P-value = 0.0373), presence of diabetes (P-value = 0.0199), balding pattern (P-value = 0.0038), current sexual activity, erectile dysfunction (ED) (P-value < 0.0001), frequent aspirin usage (P-value = 0.0003) and PSA levels (P-value < 0.0001) (Additional file 3: Table S2). Adjusted multiple logistic regression (Figure 4 and Additional file 3: Table S2) indicates a marginally increased risk of prostate cancer in the Venda population relative to the distribution of cases in the reference group Tsonga (OR = 1.81 95%CI 1.01-3.29; P-value = 0.0473), and relative to men who did not report any balding, men with a combination frontal/vertex balding pattern have a significantly greater risk of prostate cancer (OR = 1.60 95%CI 1.13-2.28; P-value = 0.0087). The association was maintained between prostate cancer risk and a family history of cancer (OR = 2.60 95%CI 1.24-5.94; P-value = 0.0155), presence of diabetes (OR = 1.83 95%CI 1.13-3.01; P-value = 0.0161), present sexual activity (OR = 0.48 95%CI 0.34-0.68; P-value < 0.0001), ED (OR = 1.53 95%CI 1.07-2.18; P-value = 0.0187) and aspirin usage (OR = 1.68 95%CI 1.20-2.37; P-value = 0.0026). Finally, we observe an increased risk of prostate cancer in men with PSA levels ≥10 μg/L, compared to those with a PSA < 4.0 μg/L (P-values < 0.0001).

Case-only analysis (Additional file 4: Table S3 and Additional file 5: Table S4) revealed several associations between variables and markers of aggressive disease including Gleason score and tumor grade. The risk ratio of having a Gleason score > 7 relative to <7 is decreased in Venda compared to the reference group Tsonga (RR = 0.36 95%CI 0.15-0.89; P-value = 0.0267), decreased for cases collected from the more urban SBAH/MEDUNSA relative to Polokwane (RR = 0.30 95%CI 0.12-0.74; P-value = 0.0088), decreases with increasing age (RR = 0.98 95%CI 0.94-0.99; P-value = 0.0137), is decreased in men who report a vertex only balding pattern (RR = 0.30 95%CI 0.10-0.95; P-value = 0.0400) and men with a higher Gleason score are less likely to be sexually active (RR = 0.50 95%CI 0.29-0.88; P-value = 0.0154). Conversely, men with a Gleason score > 7 have an increased risk of developing male breasts (RR = 1.98 95%CI 1.13-3.49; P-value = 0.0176). We also observe a decreased risk of a history of acne (RR = 0.15 95%CI 0.02-0.90; P-value = 0.0379), a decreased likelihood of present sexual activity (RR = 0.49 95%CI 0.25-0.97; P-value = 0.0395) and an increased risk of suffering ED (RR = 1.09 95%CI 0.01-1.20; P-value = 0.0322) in case samples with a Gleason score =7 compared to <7. Although few observations were significantly associated with tumor grade (possibly due to reduced sample size), we did observe an increased relative risk of being diagnosed with moderate tumor grade compared to a well differentiated tumor for men with a family history of prostate cancer (RR = 2.90 95%CI 1.14-7.43; P-value = 0.0260) and a decreased risk in men who reported balding at a relatively younger age of 30–39 yrs compared to ≥70 yrs (RR = 0.12 95%CI 0.01-0.98; P-value = 0.0477). Alternatively, later onset of ED was associated with an increased risk of both moderate (RR = 1.13 95%CI 1.03-1.25; P-value = 0.0122) and poorly (RR = 1.13 95%CI 1.00-1.26; P-value = 0.0477) differentiated tumors compared to well differentiated. Men with a PSA level ≥100 μg/L were at an increased risk of having a poorly differentiated tumor (RR = 6.84 95%CI 1.28-36.56; P-value = 0.0246).

## Discussion

One of the most significant risk factors associated with prostate cancer is an African ancestry. An admixture study of prostate cancer within African Americans led to the identification of the 8q24 prostate cancer susceptibility locus [29], arguably one of the most significant prostate cancer risk loci identified to date [30]. Prostate cancer association studies within African Americans are becoming more prevalent yet the impact of risk alleles within non-admixed African populations has been largely overlooked, mainly due to logistic issues associated with establishing African-based prostate cancer studies. We test for the first time within a unique collection of Southern Bantu prostate cancer cases as well as geographically and age matched controls, the predictive power of previously defined prostate cancer risk alleles as well as several modifiable and un-modifiable epidemiological factors.

A major issue surrounding current diagnostic procedures is the inability to determine clinically relevant prostate cancer cases from indolent/benign prostatic disease. Thus, although it could be argued that patients diagnosed with BPH are not true, disease free controls, the inclusion of these individuals in the SAPCS control population may serve to eliminate possible associations with irrelevant disease status. Additionally, a study of 5068 men concluded that presence of BPH does not increase prostate cancer risk [31], while BPH is generally formed in transitional/central zone of the prostate gland, while prostate cancer within the peripheral zone [32]. As presented elsewhere [Tindall et al., BJC, submitted], the SAPCS is biased towards a more aggressive prostate cancer phenotype, demonstrated by frequency of extreme serum PSA levels and Gleason scores, compared to White and Black prostate cancer sufferers within the USA. Lack of routine PSA testing, medical infrastructure and increased use of traditional healers all contribute to symptomatic presentation and bias towards aggressive disease (while controlling for age at presentation) compared to current studies based on western practices and indolent disease presentation. The SAPCS therefore provides a unique alternative resource to study the impact of known genetic and epidemiological factors driving aggressive prostate cancer disease within Africa.

Initial association analysis of 46 SNPs within the SAPCS revealed from 38 informative alleles a significant association (un-adjusted) with six variants and prostate cancer risk. After stage 2 analysis, we were able to replicate a significant association (un-adjusted) with only three of the previously described SNPs, namely rs6983561, rs1859962, and rs13254738. One must caution however that our power to detect statistical significance is hindered by a relatively small sample size, to between 32 and 44%. A case group >1,000 subjects would be required to achieve 80% power to detect a statistically significant (P-value < 0.05) OR ≥ 1.4 with MAF ≥ 0.2 in single hypothesis testing. Difficulties related to achieving highly significant associations with GWAS defined prostate cancer risk alleles in African populations has however been discussed previously [33]. As well as requiring large sample sizes to detect significant associations in GWAS, low levels of linkage disequilibrium in African populations may contribute to weak associations between causal variants and SNPs that are genotyped on GWAS platforms. Regardless, further validation in a larger study is required to improve power and more confidently reject the null hypothesis. We further suggest that some of the significant variants (rs6983561 and rs1859962) may be associated with clinical characteristics of prostate cancer including serum PSA levels (rs6983561) and tumor grade (rs6983561 and rs1859962). These results may be indicative that there is a genetic contribution to aggressive disease phenotype observed in these individuals. Although efforts to decipher aggressive from indolent disease have thus far shown limited success, recent reports have supported an inherited component to poorer prognosis [34,35]. Advantages of using germline genetic markers to decipher these individuals include, ease and accuracy of testing, as well as being constant throughout a life-time (non-age-dependent), hence always preceding disease and facilitating early intervention. As no variants tested in this study remained independently predictive after adjusting for multiple testing and combined within a genetic risk model showed no improvement on the predictive capability of serum PSA testing, highlights the need for independent prostate cancer genetic marker identification within the context of Africa.

Although there has thus far been limited success in identifying demographic, lifestyle or environmental influences on prostate cancer predisposition, the multi-faceted nature of this disease is indisputable. These factors have as yet not been explored within the context of Africa. We provide evidence in this study for potential drivers of prostate cancer risk within the SAPCS. While a family history of prostate cancer has been associated with increased risk for a positive diagnosis for prostate cancer within the USA [36], a familial link has previously been attributed to increased screening in men with a family history of the disease, which may contribute to this observed association. Alternatively, results from the REDUCE study, which boasts minimal screening bias, reported a geographically-dependent association between prostate cancer and a family history of the disease, yet a significant association, regardless of geographic location, was observed between prostate cancer and a family history of prostate and/or breast cancer. In our study risk was similarly not specifically attributed to a family history of prostate cancer, but rather a history of any cancer [37]. Where an Australian-based study reported an association between prostate cancer and a vertex only male balding pattern [38], the SAPCS showed a significant association with a combination of vertex and frontal balding, although age at onset was not a significant factor. While diabetes mellitus increases risk for most human cancers (reviewed in [39]), the impact on prostate cancer appears largely protective [40,41], however, an increased over-all mortality rate has been observed in prostate cancer patients with diabetes compared to those without [42]. Further complicating the assessment of this interaction is a lack of differentiating type I and type II diabetes and the potential effect of diabetes medications on prostate cancer outcome [43]. In our study of aggressive prostate cancer disease we observed a significant increased risk associated with pre-existing diabetes. In line with an Australian-based study, which correlated increased ejaculation frequency (especially early in life), with reduced prostate cancer risk [44] we show a significant protective effect of increased sexual activity and an inverse correlation with erectile dysfunction in the SAPCS. Although no association with the presence of STDs was observed, we cannot exclude that increased ejaculation, associated with sexual activity (or inversely associated with erectile dysfunction), may not be driving protection as a result of pathogenic shedding, specifically within an environment where pathogenic diseases are significant health concerns. Compared to a recent report that suggests a decreased risk of prostate cancer associated with regular use of aspirin (but not with alternative non-steroidal anti-inflammatory drugs), [45]], frequent aspirin use within the SAPCS was inversely correlated with prostate cancer. This disparity may be impacted by the generic employment of the term aspirin, often used to refer to any form of headache medicine, including paracetemol, which exhibits very minor anti-inflammatory activity. A unique aspect of the SAPCS is the inclusion of both rural and more urbanized clinic locations. The significance of the observed increased prostate cancer risk associated with men from the Venda ethnolinguistic classification requires further investigation based on genetic and/or environmental drivers. A potential significant environmental implication for the observed association may be based on almost 70 years of dichlorodiphenyltrichloroethane (DDT) spraying for malaria control in Venda households [46] and previous controversial association with urogenital birth defects [47]. Interestingly, men within a health or education related occupation were more likely to be diagnosed with prostate cancer. The latter could be a direct consequence of increased access and adoption of western medical practices. Not surprisingly, men with extreme PSA levels (≥20 ng/μl) were also at an increased risk of disease. Although we present some evidence of association between aggressive disease phenotypes (high Gleason score and tumor grade) and epidemiological characteristics, limited study numbers (345 total cases with known Gleason score and 305 cases with known tumor grade) reduces the impact of any findings. Our results do however warrant further investigation into associations with specific population groups, geographic location, age of prostate cancer onset, family history of cancer, sexual capacity, balding pattern and age of balding, as well as development of male breasts and acne.

## Conclusions

In an attempt to use individual genetic profiles of the SAPCS population to determine prostate cancer occurrence, we failed to show a significant predictive power alone or improve correlation in combination with the current serum PSA testing method. From these results we suggest the current content of prostate cancer GWAS is inadequate for predicting prostate cancer in the Southern African Bantu study population and not suitable for implementation in a clinical setting. The nature of previously defined risk variants, which are thought to typically represent indirect associations (often in gene deserts) and a minimal effect size (small ORs), likely contributes to the limited impact on disease prediction we and others have thus far observed. Current methods of GWAS are based on the principle of LD, which is considered disadvantageous in African populations known to exhibit high levels of genetic diversity and low levels of LD [48]. Increased haplotype diversity, is however considered beneficial for the purpose of fine-mapping and identification of causal variants, potentially making this cohort a valuable resource for defining causal variants. It is likely that new approaches in statistical imputation and next generation sequencing technologies will not only contribute to defining true causal variants, but also better define high-risk population groups to help identify population specific risk loci. As we move into the era of personalized medicine, large scale sequencing efforts will help improve current GWAS platforms and identification of prognostic markers via the development of population specific and disease specific genetic profiles. Furthermore, we cannot ignore the potential impact of demographic and lifestyle influences on prostate cancer occurrence. These pilot analyses are directing further investigative efforts as the study leaders focus on increasing the study numbers to achieve optimal study power. The validation of known, specifically modifiable, risk factors calls for immediate replication in parallel studies from within Africa. In summary, the SAPCS provides a unique resource not only to identify genetic determinants associated with an African ancestry but also gene-environment interactions in a collection of clinically relevant (aggressive) prostate cancer cases, minimizing the impact of indolent disease often dominant in studies from developed nations.

## Abbreviations

SAPCS: Sothern African Prostate Cancer Study; GWAS: Genome-wide association study; SBAH: Steve Biko Academic Hospital; MEDUNSA: Medical University of South Africa; GRS (cGRS: wGRS), Genetic risk score (count GRS, weighted GRS); ROC: Receiver operating characteristic curve; AUC: Area under the curve; ED: Erectile dysfunction; LD: Linkage disequilibrium.

## Competing interests

The authors declare that they have no competing interests.

## Authors’ contributions

EAT performed genotyping, data analysis and interpretation. PAV, MSRB and VMH conceived the study design and received seed funding. MSRB, LRM, SVZ and AMS are participating urologists responsible for study recruitment and data collection. EAT, MSRB, PAV and VMH are responsible for collating and maintaining the database. VMH established and maintains the SAPCS webpage. EAT and VMH drafted the manuscript. All authors read and approved the final manuscript.

## Pre-publication history

The pre-publication history for this paper can be accessed here:

http://www.biomedcentral.com/1471-2490/13/74/prepub

## Supplementary Material

Additional file 1: Table S1 Reported prostate cancer risk alleles achieving genome-wide significance (P value < 10^-6^) and stage 1 genotype association results. Click here for file

Additional file 2: Figure S1 Genetic risk models 1–3 for 3, 6 and 38 SNPs. (a) Genetic risk Model 1: Count GRS (cGRS) is defined as the total sum of risk alleles present at each SNP (cGRS = ∑risk alleles). (b) Genetic risk Model 2: weighted GRS (wGRS) considers both the number of risk alleles and the OR attributed to that SNP (wGRS = LN OR∑risk alleles). (c) Genetic risk Model 3: Risk relative to the average population is defined as the genotypic risk (OR^n risk alleles) divided by the average population risk (∑population frequency x genotypic risk) and calculated for each genotype combination. All models were calculated using 38 SNPs (d), 6 SNPs that reached statistical significance in stage 1 (e) and 3 SNPs significant in stage 2 (f). Population specific study ORs and allele frequencies were used to calculate GRSs. For genetic risk Models 2 and 3 (b) and (c) the 6 SNP and 38 SNP combinations significantly improved predictive power compared to 3 SNPs only. For Model 2 the difference between AUC = 0.035 (95% CI 0.005-0.065, P = 0.0208) and 0.065 (95% CI 0.015-0.116, P = 0.0117) for 6 and 38 SNPs respectively. For Model 3, the difference between AUC = 0.047 (95% CI 0.005-0.088, P = 0.0282) and 0.093 (95% CI 0.027-0.159, P = 0.0061) for 6 and 38 SNPs respectively. There was no significant difference within each model for any of the *n* SNP combinations (d) to (f). Click here for file

Additional file 3: Table S2 SAPCS study characteristics and association with prostate cancer risk. Click here for file

Additional file 4: Table S3 Case-only analysis for association between epidemiological measures and Gleason score. Click here for file

Additional file 5: Table S4 Case-only analysis for association between epidemiological measures and tumor grade. Click here for file
