# Supplementary material for: Addressing the contribution of previously described genetic and epidemiological risk factors associated with increased prostate cancer risk and aggressive disease within men from South Africa
Source: BMC Urol. 2013 Dec 29;13:74. doi: 10.1186/1471-2490-13-74 (PMC3882498; doi:10.1186/1471-2490-13-74)
Supplement: Additional file 1: Table S1 — Reported prostate cancer risk alleles achieving genome-wide significance (P value < 10-6) and stage 1 genotype association results. [file 1471-2490-13-74-S1.docx]

| **Table S1.** Reported prostate cancer risk alleles achieving genome-wide significance (P value<10^-6^) and stage 1 genotype association results. | | | | | | | | | | | | | | | | |  |
| --- | --- | --- | --- | --- | --- | --- | --- | --- | --- | --- | --- | --- | --- | --- | --- | --- | --- |
|  |  |  | Discovery study (GWAS+targeted)^†^ | | | Replication^¶^ | | | This study stage 1 (179 cases, 144 controls) | | | | | | | |  |
| Locus | SNP ID  (risk allele) | Gene* | Popn^‡^ | OR | Platform; n SNPs^§^  (GWAS only) | Popn^‡^ | | | MAF  cases | | MAF cont | | OR (95% CI) ^¥^ | | P-value^¥^ | | Q-value^£^ |
| SNPs assessed in this study – Publication dates 06/2006-04/2010 | | | | | | | | | | | | | | | | |  |
| 2p15 | rs721048 (A) | *EHBP1* | EU^1^ | 1.23 | Illumina, 310,520 | - | | | 0.003 | | 0.007 | | 0.38 (0.03-4.25) | | | 0.5826 | 0.5566 |
| 2p21 | **rs1465618** (A) | *THADA* | EU^2^ | 1.16 | Illumina, 541,129 | - | | | **0.118** | | **0.069** | | **1.80 (1.02-3.16)** | | | **0.0407** | 0.2017 |
| 2q31 | rs12621278 (G) | *ITGA6* | EU^2^ | 0.72 | Illumina, 541,129 | - | | | 0 | | 0 | | - | | | - | - |
| 3p12 | rs2660753 (T) | - | EU^3^ | 1.35 | Illumina, 541,129 | - | | | 0.438 | | 0.391 | | 1.22 (0.88-1.68) | | | 0.2536 | 0.3696 |
| 3q21 | rs10934853 (A) | *EEFSEC* | EU^4^ | 1.12 | Illumina, 310,520 | - | | | 0.169 | | 0.150 | | 1.15 (0.75-1.78) | | | 0.5842 | 0.5566 |
| 4q22 | rs17021918 (T) | *PDLIM5* | EU^2^ | 0.86 | Illumina, 541,129 | - | | | 0.247 | | 0.259 | | 0.94 (0.65-1.35) | | | 0.7810 | 0.5988 |
|  | rs12500426 (A) | *PDLIM5* | EU^2^ | 1.13 | Illumina, 541,129 | - | | | *Failed* | | - | | - | | | - | - |
| 4q24 | rs7679673 (A) | *FLJ20032* | EU^2^ | 0.86 | Illumina, 541,129 | - | | | 0.289 | | 0.237 | | 1.31 (0.91-1.88) | | | 0.1472 | 0.2631 |
| 6q25 | rs9364554 (T) | *SLC22A3* | EU^3^ | 1.23 | Illumina, 541,129 | - | | | 0.011 | | 0.004 | | 3.10 (0.34-27.92) | | | 0.3941 | 0.4881 |
| 7q21 | rs6465657 (C) | *LMTK2* | EU^3^ | 1.21 | Illumina, 541,129 | - | | | 0.014 | | 0.026 | | 0.54 (0.17-1.73) | | | 0.3810 | 0.4881 |
| 8p21 | rs2928679 (T) | *NKX3.1* | EU^2^ | 1.14 | Illumina, 541,129 | - | | | 0.294 | | 0.303 | | 0.96 (0.68-1.35) | | | 0.8604 | 0.5988 |
|  | rs1512268 (A) | *NKX3.1* | EU^2^ | 1.18 | Illumina, 541,129 | JPT^5^ | | | 0.340 | | 0.277 | | 1.34 (0.95-1.89) | | | 0.1000 | 0.2248 |
| 8q24 (1) | rs1447295 (A) | - | EU^6^ | 1.72 | - | EU ^7-10^; JPT^5^ | | | 0.404 | | 0.398 | | 1.03 (0.75-1.42) | | | 0.8702 | 0.5988 |
|  | **rs4242382** (A) | - | EU^7^ | 1.40 | - | EU^3, 11^ | | | **0.253** | | **0.325** | | **0.70 (0.50-0.996)** | | | **0.0503** | 0.2077 |
|  | **rs10090154 (T)** | - | EU^12^ | 1.67 | Illumina, 541,129 | ME^13^ | | | **0.138** | | **0.226** | | **0.55 (0.36-0.83)** | | | **0.0044** | 0.1090 |
| 8q24 (2) | rs1016343 (T) | - | EU^12^ | 1.29 | Illumina, 541,129 | EU^3^; JPT^5^ | | | *Failed* | | - | | - | | | - | - |
|  | rs13252298 (G) | - | EU^12^ | 0.82 | Illumina, 541,129 | - | | | 0.028 | | 0.022 | | 1.31 (0.47-3.64) | | | 0.7996 | 0.5988 |
|  | **rs6983561 (C)** | - | EU^12^ | 1.79 | Illumina, 541,129 | JPT^14^; ME^13^; AA^15, 16^ | | | **0.478** | | **0.372** | | **1.54 (1.12-2.13)** | | | **0.0094** | 0.1164 |
|  | rs16901979 (A) | - | EU^9^ | 1.79 | Illumina, 316,515 | ME^13^; AA^15^; JPT^14^; CH^17^; EU^18^ | | | 0.443 | | 0.368 | | 1.37 (0.99-1.89) | | | 0.0596 | 0.2109 |
|  | rs7841060 (G) | - | EU^18^ | 1.22 | - | - | | | 0.421 | | 0.438 | | 0.94 (0.68-1.29) | | | 0.7448 | 0.5988 |
|  | rs4871008 (C) | - | EU^18^ | 1.14 | - | - | | | 0.194 | | 0.188 | | 1..04 (0.70-1.56) | | | 0.9185 | 0.6150 |
|  | rs6470494 (T) | - | EU^18^ | 1.14 | - | - | | | 0.304 | | 0.328 | | 0.89 (0.64-1.25) | | | 0.5440 | 0.5566 |
|  | **rs13254738 (C)** | - | ME^13^ | 1.26 | - | AA^15^ ^16^ | | | **0.331** | | **0.416** | | **0.70 (0.50-0.96)** | | | **0.0306** | 0.2017 |
|  | Bd11934905^ | - | AA^13^ | 2.45 | - | - | | | 0.065 | | 0.036 | | 1.82 (0.85-3.90) | | | 0.1487 | 0.2631 |
| 8q24 (3) | rs6983267 (G) | - | EU^10^ | 1.22 | Illumina; 538,548 | EU^3, 7, 11, 12^; ME^13^ | | | 0 | | 0 | | - | | | - | - |
|  | rs7000448 (T) | - | ME^13^ | 1.26 | - | - | | | *Failed* | | - | | - | | | - | - |
| 8q24 (4) | rs620861 (C)  (T) | - | EU^12^  EU^18^ | 0.84  1.16 | Illumina, 541,129 | - | | | *Failed* | | - | | - | | | - | - |
|  | rs445114 (T) | - | EU^4^  EU^18^ | 1.21  1.16 | Illumina, 310,520 | - | | | 0.331 | | 0.281 | | 1.27 (0.90-1.79) | | | 0.1925 | 0.3179 |
|  | rs16902104 (T) | - | EU^4^ | 1.14 | - | - | | | 0.093 | | 0.079 | | 1.19 (0.68-2.09) | | | 0.5727 | 0.5566 |
| 8q24 (5) | rs10086908 (C) | - | EU^12^ | 0.84 | Illumina, 541,129 |  | | | 0.227 | | 0.288 | | 0.72 (0.51-1.04) | | | 0.0819 | 0.2248 |
| 10q11 | rs10993994 (T) | *MSMB* | EU^3^  EU^11^ | 1.25 | Illumina, 541,129  Illumina; 527,869 | EU^19-21^; JPT^5^ | | | *Failed* | | - | | - | | | - | - |
| 10q26 | rs4962416 (C) | *CTBP2* | EU^11^ | 1.09 | Illumina; 527,869 | - | | | 0.205 | | 0.153 | | 1.42 (0.94-2.16) | | | 0.0974 | 0.2248 |
| 11p15 | rs7127900 (A) | - | EU^2^ | 1.26 | Illumina, 541,129 | - | | | 0.474 | | 0.485 | | 0.96 (0.70-1.31) | | | 0.8088 | 0.5988 |
| 11q13 | rs7931342 (T) | - | EU^3^ | 0.82 | Illumina, 541,129 | EU^19^ | | | 0.118 | | 0.095 | | 1.28 (0.76-2.14) | | | 0.3682 | 0.4881 |
|  | rs10896449 (G) | - | EU^11^ | 1.22 | Illumina; 527,869 | EU^22, 23^ | | | 0.213 | | 0.201 | | 1.08 (0.73-1.60) | | | 0.7665 | 0.5988 |
|  | rs11228565 (A) | - | EU^4^ | 1.23 | Illumina, 310,520 | - | | | 0.051 | | 0.044 | | 1.16 (0.55-2.46) | | | 0.8507 | 0.5988 |
| 17q12 (1) | rs7501939 (C) | *HNF1B* | EU^24^ | 1.19 | Illumina; 310,520 | EU^3, 25^; JPT^5^ | | | 0.441 | | 0.420 | | 1.09 (0.79-1.50) | | | 0.6267 | 0.5750 |
|  | rs4430796 (A) | *HNF1B* | EU^24^ | 1.22 | Illumina; 310,520 | EU^11, 25-27^ | | | 0.275 | | 0.215 | | 1.38 (0.96-2.00) | | | 0.0946 | 0.2248 |
| 17q21 | rs7210100 (A)^ | *ZNF652* | AA^28^ | 1.51 | Illumina; 1,047,198 | - | | | 0.041 | | 0.022 | | 1.89 (0.87-4.11) | | | 0.1089 | 0.2248 |
| 17q24 | **rs1859962 (G)** | - | EU^24^ | 1.20 | Illumina; 310,520 | EU^3^ | | | **0.270** | | **0.197** | | **1.50 (1.03-2.20)** | | | **0.0379** | 0.2017 |
| 19q13 | rs2735839 (A) | *KLK2/3* | EU^3^ | 0.83 | Illumina, 541,129 | EU^29^ | | | 0.382 | | 0.380 | | 1.01 (0.73-1.40) | | | 1.0000 | 0.6519 |
|  | rs887391 (T) | - | EU^30^ | 1.15 | Affymetrix; 260,852 | - | | | 0.418 | | 0.391 | | 1.12 (0.81-1.55) | | | 0.5130 | 0.5566 |
|  | rs8102476 (C) | *PPP1R14A* | EU^4^ | 1.12 | Illumina, 310,520 | - | | | *Failed* | | - | | - | | | - | - |
| 22q13 | rs5759167 (T) | *BIK* | EU^2^ | 0.86 | Illumina, 541,129 | - | | | 0.192 | | 0.168 | | 1.18 (0.78-1.78) | | | 0.4659 | 0.5496 |
| Xp11 | rs5945572 (A) | *NUDT10/11* | EU^1^ | 1.15 | Illumina, 310,520 | - | | | 0.326 | | 0.277 | | 1.26 (0.89-1.78) | | | 0.2216 | 0.3431 |
|  | rs5945619 (C) | *NUDT10/11* | EU^3^ | 1.19 | Illumina, 541,129 | EU^19^ | | | 0.446 | | 0.460 | | 0.94 (0.69-1.30) | | | 0.7462 | 0.5988 |
| SNPs not assessed in this study – Publication dates 05/2010-10/2012 | | | | | | | | | | | | | | | | |  |
| 2p11 | rs10187424 (G) | *GGCX* | EU^31^ | 0.90 | Illumina; 541,129 | | - |  | |  | |  | |  | | |  |
|  | rs2028898 (C) | *GGCX* | JPT^32^ | 1.15 | Illumina; 510,687 | |  |  | |  | |  | |  | | |  |
| 2p24 | rs13385191 (G) | *C2orf43* | JPT^5^ | 1.15 | Illumina; 510,687 | | - |  | |  | |  | |  | | |  |
| 2q37 | rs2292884 (G) | *MLPH* | EU^33^ | 1.14 | Illumina; 571,243 | | - |  | |  | |  | |  | | |  |
|  | rs7584330 (C) | *MLPH* | EU^31^ | 1.12 | Illumina; 541,129 | | - |  | |  | |  | |  | | |  |
| 3p11 | rs7629490 (T) | - | EU^33^ | 1.15 | Illumina; 571,243 | | - |  | |  | |  | |  | | |  |
|  | rs2055109 (C) | - | JPT^32^ | 1.20 | Illumina; 510,687 | |  |  | |  | |  | |  | | |  |
| 3p12 | rs17023900 (G) | - | LA^34^ | 1.58 | Illumina, 541,129 | |  |  | |  | |  | |  | | |  |
| 3q23 | rs6763931 (T) | *ZBTB38* | EU^31^ | 1.10 | Illumina; 541,129 | | - |  | |  | |  | |  | | |  |
| 3q26 | rs10936632 (C) | *CLDN11*/*SKIL* | EU^31^ | 0.87 | Illumina; 541,129 | | - |  | |  | |  | |  | | |  |
| 5p12 | rs2121875 (G) | *FGF10* | EU^31^ | 1.11 | Illumina; 541,129 | | - |  | |  | |  | |  | | |  |
| 5p15 | rs12653946 (T) | - | JPT^5^ | 1.26 | Illumina; 510,687 | | CH^35^ |  | |  | |  | |  | | |  |
|  | rs2242652 (A) | *TERT* | EU^31^ | 0.81 | Illumina; 541,129 | | - |  | |  | |  | |  | | |  |
| 6p21 | rs1983891 (T) | *FOXP4* | JPT^5^ | 1.15 | Illumina; 510,687 | | - |  | |  | |  | |  | | |  |
|  | rs130067 (G) | *CCHCR1* | EU^31^ | 1.12 | Illumina; 541,129 | | - |  | |  | |  | |  | | |  |
| 6q22 | rs339331 (T) | *RFX6/GPRC6A* | JPT^5^ | 1.22 | Illumina; 510,687 | | - |  | |  | |  | |  | | |  |
|  | rs12202378 (T) | *RFX6* | AA^16^ | 1.25 | - | | - |  | |  | |  | |  | | |  |
| 6q25 | rs651164 (A) | *SLC22A1* | EU^33^ | 0.85 | Illumina; 571,243 | | - |  | |  | |  | |  | | |  |
| 7p15 | rs10486567 (G) | *JAZF1* | EU^36^ | 1.18 |  | | - |  | |  | |  | |  | | |  |
| 8q24 (1) | rs7017300 | - | EU^7^ |  | - | | - |  | |  | |  | |  | | |  |
|  | rs7837688 | - | EU^7^ |  | - | | - |  | |  | |  | |  | | |  |
| 8q24 (2) | rs1456316 (T) | - | EU^18^ | 1.12 | - | | - |  | |  | |  | |  | | |  |
|  | rs1456315 (T) | - | AA^16^ | 1.23 | *-* | | - |  | |  | |  | |  | | |  |
|  | rs6987409 (T) | - | AA^16^ | 1.42 | *-* | | - |  | |  | |  | |  | | |  |
| 8q24 (4) | rs587948 (G) | - | EU^18^ | 1.13 | - | | - |  | |  | |  | |  | | |  |
| 10q26 | rs2252004 (G) | - | JPT^32^ | 1.16 | Illumina; 510,687 | |  |  | |  | |  | |  | | |  |
|  | rs11199874 (G) | - | EU^37^ | 1.42 | Affymetrix; 443,816 | |  |  | |  | |  | |  | | |  |
|  | rs10788165 (T)  rs10749408 (T) | -  - | EU^37^  EU^37^ | 1.34  1.26 | Affymetrix; 443,816  Affymetrix; 443,816 | |  |  | |  | |  | |  | | |  |
| 11q12 | rs1938781 (C) | *FAM111A/B* | JPT^32^ | 1.16 | Illumina; 510,687 | |  |  | |  | |  | |  | | |  |
| 11q13 | rs1128580 (C) | *DENR* | AA^16^ | 1.31 | - | | - |  | |  | |  | |  | | |  |
| 12q13 | rs902774 (A) | - | EU^33^ | 1.17 | Illumina; 571,243 | | - |  | |  | |  | |  | | |  |
|  | rs10875943 (C) | - | EU^31^ | 1.10 | Illumina; 541,129 | | - |  | |  | |  | |  | | |  |
| 13q22 | rs9600079 (T) | - | JPT^5^ | 1.18 | Illumina; 510,687 | | - |  | |  | |  | |  | | |  |
| 15q21 | rs4775302 (A)  rs1994198 (T) | -  - | EU^37^  EU^37^ | 1.41  1.34 | Affymetrix; 443,816  Affymetrix; 443,816 | |  |  | |  | |  | |  | | |  |
| 15q25 | rs7175701 (C) | *IL-16* | AA^38^ | 1.98 | - | |  |  | |  | |  | |  | | |  |
| 17q12 (1) | rs2005705 (T) | *HNF1B* | EU^25^ | 0.81 | - | | - |  | |  | |  | |  | | |  |
|  | rs7405696 (C) | *HNF1B* | EU^25^ | 1.23 | - | | - |  | |  | |  | |  | | |  |
|  | rs4239217 (G) | *HNF1B* | EU^25^ | 0.84 | - | | - |  | |  | |  | |  | | |  |
|  | rs757210 (A) | *HNF1B* | EU^25^ | 0.84 | - | | - |  | |  | |  | |  | | |  |
|  | rs3760511 (C) | *HNF1B* | EU^25^ | 1.19 | - | | - |  | |  | |  | |  | | |  |
|  | rs3744763 (C) | *HNF1B* | EU^25^ | 0.89 | - | | - |  | |  | |  | |  | | |  |
| 17q12 (2) | rs4794758 (T) | *HNF1B* | EU^25^ | 0.86 | - | | - |  | |  | |  | |  | | |  |
|  | rs11649743 (G) | *HNF1B* | EU^27^ | 1.22 | - | | EU^25^ |  | |  | |  | |  | | |  |
| 19q13 | rs17632542 (C) | *KLK2/3* | EU^29^ | 0.59 | - | | - |  | |  | |  | |  | | |  |
|  | rs266849 (G) | *KLK2/3* | EU^3^ | 0.79 | Illumina, 541,129 | | EU^29^ |  | |  | |  | |  | | |  |
|  | rs1058205 (C) | *KLK3* | EU^29^ | 0.75 | - | | - |  | |  | |  | |  | | |  |
| 22q13 | rs9623117 (C) | *TNRC6B* | EU^39^ | 1.18 | Affymetrix+Illumina; ~2million imputed | | - |  | |  | |  | |  | | |  |
| Xq12 | rs5919342 (G) | - | EU^31^ | 0.92 | Illumina; 541,129 | | - |  | |  | |  | |  | | |  |
| MAF = minor allele frequency; OR = odds ratio  * Gene within the linkage disequilibrium block defined by the associated variant  ^†^ Discovery study: First reports of each SNP reaching genome-wide significance (P-value<10^-6^) and include GWAS and targeted studies. GWAS (Genome-wide association studies) may combine data from multiple stages. Stage 1 is designed to capture all common genetic variation across entire genome in an un-biased manner. Subsequent GWAS stages may be influenced by stage 1 results. Targeted and/or fine-mapping studies first to report genome-wide significance are listed. These studies focus on specific regions/variants.  ^‡^ Popn (population): EU (European); AA (African American); LA (Latin American); JPT (Japanese); CH (Chinese); ME (Multi-Ethnic: AA [29%], JPT [27%], Latino [24%], EU [17%] and native Hawaiian [4%]).  ^§^ Genotyping platform manufacturer and number (n) SNPs to pass study QC in stage 1 of GWAS.  ^¶^ Replication: Follow-on studies independently achieving genome wide significance threshold for each SNP.  ^¥^ P-value determined by Fischer’s exact test comparing allele distribution in cases versus controls. Allelic OR and 95% CI were calculated using logistic regression models. **Bolded** text indicate P-values (P≤0.05) in stage 1 analysis of this study.  ^£^ Q-values correct for false discovery rate from multiple testing.  ^ Bd11934905 and rs7210100 were selected for genotype analysis in this study due to significant associations observed specifically in African American populations. Bd11934905 did not achieve genome-wide significance (P=1.5x10^-4^) in the discovery target study. rs7210100 did achieve genome-wide significance in the discovery GWAS (first AA GWAS; P=3.4x10^-13^), but was published after the pre-defined publication deadline (April, 2010) and therefore not genotyped in the initial stage 1 analysis of this study. | | | | | | | | | | | | | | | | |  |

**Table S1 References**

1. Gudmundsson J, Sulem P, Rafnar T, et al. Common sequence variants on 2p15 and Xp11.22 confer susceptibility to prostate cancer. *Nat Genet* 2008 Mar;**40**(3): 281-3.

2. Eeles RA, Kote-Jarai Z, Al Olama AA, et al. Identification of seven new prostate cancer susceptibility loci through a genome-wide association study. *Nat Genet* 2009 Oct;**41**(10): 1116-21.

3. Eeles RA, Kote-Jarai Z, Giles GG, et al. Multiple newly identified loci associated with prostate cancer susceptibility. *Nat Genet* 2008 Mar;**40**(3): 316-21.

4. Gudmundsson J, Sulem P, Gudbjartsson DF, et al. Genome-wide association and replication studies identify four variants associated with prostate cancer susceptibility. *Nat Genet* 2009 Oct;**41**(10): 1122-6.

5. Takata R, Akamatsu S, Kubo M, et al. Genome-wide association study identifies five new susceptibility loci for prostate cancer in the Japanese population. *Nat Genet* 2010 Sep;**42**(9): 751-4.

6. Amundadottir LT, Sulem P, Gudmundsson J, et al. A common variant associated with prostate cancer in European and African populations. *Nat Genet* 2006 Jun;**38**(6): 652-8.

7. Zheng SL, Sun J, Cheng Y, et al. Association between two unlinked loci at 8q24 and prostate cancer risk among European Americans. *J Natl Cancer Inst* 2007 Oct;**99**(20): 1525-33.

8. Schumacher FR, Feigelson HS, Cox DG, et al. A common 8q24 variant in prostate and breast cancer from a large nested case-control study. *Cancer Res* 2007 Apr;**67**(7): 2951-6.

9. Gudmundsson J, Sulem P, Manolescu A, et al. Genome-wide association study identifies a second prostate cancer susceptibility variant at 8q24. *Nat Genet* 2007 May;**39**(5): 631-7.

10. Yeager M, Orr N, Hayes RB, et al. Genome-wide association study of prostate cancer identifies a second risk locus at 8q24. *Nat Genet* 2007 May;**39**(5): 645-9.

11. Thomas G, Jacobs KB, Yeager M, et al. Multiple loci identified in a genome-wide association study of prostate cancer. *Nat Genet* 2008 Mar;**40**(3): 310-5.

12. Al Olama AA, Kote-Jarai Z, Giles GG, et al. Multiple loci on 8q24 associated with prostate cancer susceptibility. *Nat Genet* 2009 Oct;**41**(10): 1058-60.

13. Haiman CA, Patterson N, Freedman ML, et al. Multiple regions within 8q24 independently affect risk for prostate cancer. *Nat Genet* 2007 May;**39**(5): 638-44.

14. Yamada H, Penney KL, Takahashi H, et al. Replication of prostate cancer risk loci in a Japanese case-control association study. *J Natl Cancer Inst* 2009 Oct;**101**(19): 1330-6.

15. Chang BL, Spangler E, Gallagher S, et al. Validation of genome-wide prostate cancer associations in men of African descent. *Cancer Epidemiol Biomarkers Prev* 2011 Jan;**20**(1): 23-32.

16. Haiman CA, Chen GK, Blot WJ, et al. Characterizing genetic risk at known prostate cancer susceptibility loci in African Americans. *PLoS Genet* 2011 May;**7**(5): e1001387.

17. Liu F, Hsing AW, Wang X, et al. Systematic confirmation study of reported prostate cancer risk-associated single nucleotide polymorphisms in Chinese men. *Cancer Sci* 2011 Jul.

18. Yeager M, Chatterjee N, Ciampa J, et al. Identification of a new prostate cancer susceptibility locus on chromosome 8q24. *Nat Genet* 2009 Oct;**41**(10): 1055-7.

19. Kote-Jarai Z, Easton DF, Stanford JL, et al. Multiple novel prostate cancer predisposition loci confirmed by an international study: the PRACTICAL Consortium. *Cancer Epidemiol Biomarkers Prev* 2008 Aug;**17**(8): 2052-61.

20. Chang BL, Cramer SD, Wiklund F, et al. Fine mapping association study and functional analysis implicate a SNP in MSMB at 10q11 as a causal variant for prostate cancer risk. *Hum Mol Genet* 2009 Apr;**18**(7): 1368-75.

21. Lou H, Yeager M, Li H, et al. Fine mapping and functional analysis of a common variant in MSMB on chromosome 10q11.2 associated with prostate cancer susceptibility. *Proc Natl Acad Sci U S A* 2009 May;**106**(19): 7933-8.

22. Zheng SL, Stevens VL, Wiklund F, et al. Two independent prostate cancer risk-associated Loci at 11q13. *Cancer Epidemiol Biomarkers Prev* 2009 Jun;**18**(6): 1815-20.

23. Chung CC, Ciampa J, Yeager M, et al. Fine mapping of a region of chromosome 11q13 reveals multiple independent loci associated with risk of prostate cancer. *Hum Mol Genet* 2011 Jul;**20**(14): 2869-78.

24. Gudmundsson J, Sulem P, Steinthorsdottir V, et al. Two variants on chromosome 17 confer prostate cancer risk, and the one in TCF2 protects against type 2 diabetes. *Nat Genet* 2007 Aug;**39**(8): 977-83.

25. Berndt SI, Sampson J, Yeager M, et al. Large-scale fine mapping of the HNF1B locus and prostate cancer risk. *Hum Mol Genet* 2011 Aug;**20**(16): 3322-9.

26. Zheng SL, Sun J, Wiklund F, et al. Cumulative association of five genetic variants with prostate cancer. *N Engl J Med* 2008 Feb;**358**(9): 910-9.

27. Sun J, Zheng SL, Wiklund F, et al. Evidence for two independent prostate cancer risk-associated loci in the HNF1B gene at 17q12. *Nat Genet* 2008 Oct;**40**(10): 1153-5.

28. Haiman CA, Chen GK, Blot WJ, et al. Genome-wide association study of prostate cancer in men of African ancestry identifies a susceptibility locus at 17q21. *Nat Genet* 2011 Jun;**43**(6): 570-3.

29. Kote-Jarai Z, Amin Al Olama A, Leongamornlert D, et al. Identification of a novel prostate cancer susceptibility variant in the KLK3 gene transcript. *Hum Genet* 2011 Jun;**129**(6): 687-94.

30. Hsu FC, Sun J, Wiklund F, et al. A novel prostate cancer susceptibility locus at 19q13. *Cancer Res* 2009 Apr;**69**(7): 2720-3.

31. Kote-Jarai Z, Olama AA, Giles GG, et al. Seven prostate cancer susceptibility loci identified by a multi-stage genome-wide association study. *Nat Genet* 2011;**43**(8): 785-91.

32. Akamatsu S, Takata R, Haiman CA, et al. Common variants at 11q12, 10q26 and 3p11.2 are associated with prostate cancer susceptibility in Japanese. *Nat Genet* 2012 Apr;**44**(4): 426-9, S1.

33. Schumacher FR, Berndt SI, Siddiq A, et al. Genome-wide association study identifies new prostate cancer susceptibility loci. *Hum Mol Genet* 2011 Jul.

34. Cheng I, Chen GK, Nakagawa H, et al. Evaluating genetic risk for prostate cancer among Japanese and Latinos. *Cancer Epidemiol Biomarkers Prev* 2012 Aug.

35. Wang M, Liu F, Hsing AW, et al. Replication and cumulative effects of GWAS-identified genetic variations for prostate cancer in Asians: a case-control study in the ChinaPCa consortium. *Carcinogenesis* 2012 Feb;**33**(2): 356-60.

36. Prokunina-Olsson L, Fu YP, Tang W, et al. Refining the prostate cancer genetic association within the JAZF1 gene on chromosome 7p15.2. *Cancer Epidemiol Biomarkers Prev* 2010 May;**19**(5): 1349-55.

37. Nam RK, Zhang W, Siminovitch K, et al. New variants at 10q26 and 15q21 are associated with aggressive prostate cancer in a genome-wide association study from a prostate biopsy screening cohort. *Cancer Biol Ther* 2011 Dec;**12**(11): 997-1004.

38. Batai K, Shah E, Murphy AB, et al. Fine-mapping of IL-16 gene and prostate cancer risk in African Americans. *Cancer Epidemiol Biomarkers Prev* 2012 Aug.

39. Sun J, Zheng SL, Wiklund F, et al. Sequence variants at 22q13 are associated with prostate cancer risk. *Cancer Res* 2009 Jan;**69**(1): 10-5.
