# Supplementary material for: Addressing the contribution of previously described genetic and epidemiological risk factors associated with increased prostate cancer risk and aggressive disease within men from South Africa
Source: BMC Urol. 2013 Dec 29;13:74. doi: 10.1186/1471-2490-13-74 (PMC3882498; doi:10.1186/1471-2490-13-74)
Supplement: Additional file 2: Figure S1 — Genetic risk models 1–3 for 3, 6 and 38 SNPs. (a) Genetic risk Model 1: Count GRS (cGRS) is defined as the total sum of risk alleles present at each SNP (cGRS = ∑risk alleles). (b) Genetic risk Model 2: weighted GRS (wGRS) considers both the number of risk alleles and the OR attributed to that SNP (wGRS = LN OR∑risk alleles). (c) Genetic risk Model 3: Risk relative to the average population is defined as the genotypic risk (OR^n risk alleles) divided by the average population risk (∑population frequency x genotypic risk) and calculated for each genotype combination. All models were calculated using 38 SNPs (d), 6 SNPs that reached statistical significance in stage 1 (e) and 3 SNPs significant in stage 2 (f). Population specific study ORs and allele frequencies were used to calculate GRSs. For genetic risk Models 2 and 3 (b) and (c) the 6 SNP and 38 SNP combinations significantly improved predictive power compared to 3 SNPs only. For Model 2 the difference between AUC = 0.035 (95% CI 0.005-0.065, P = 0.0208) and 0.065 (95% CI 0.015-0.116, P = 0.0117) for 6 and 38 SNPs respectively. For Model 3, the difference between AUC = 0.047 (95% CI 0.005-0.088, P = 0.0282) and 0.093 (95% CI 0.027-0.159, P = 0.0061) for 6 and 38 SNPs respectively. There was no significant difference within each model for any of the n SNP combinations (d) to (f). [file 1471-2490-13-74-S2.pdf]

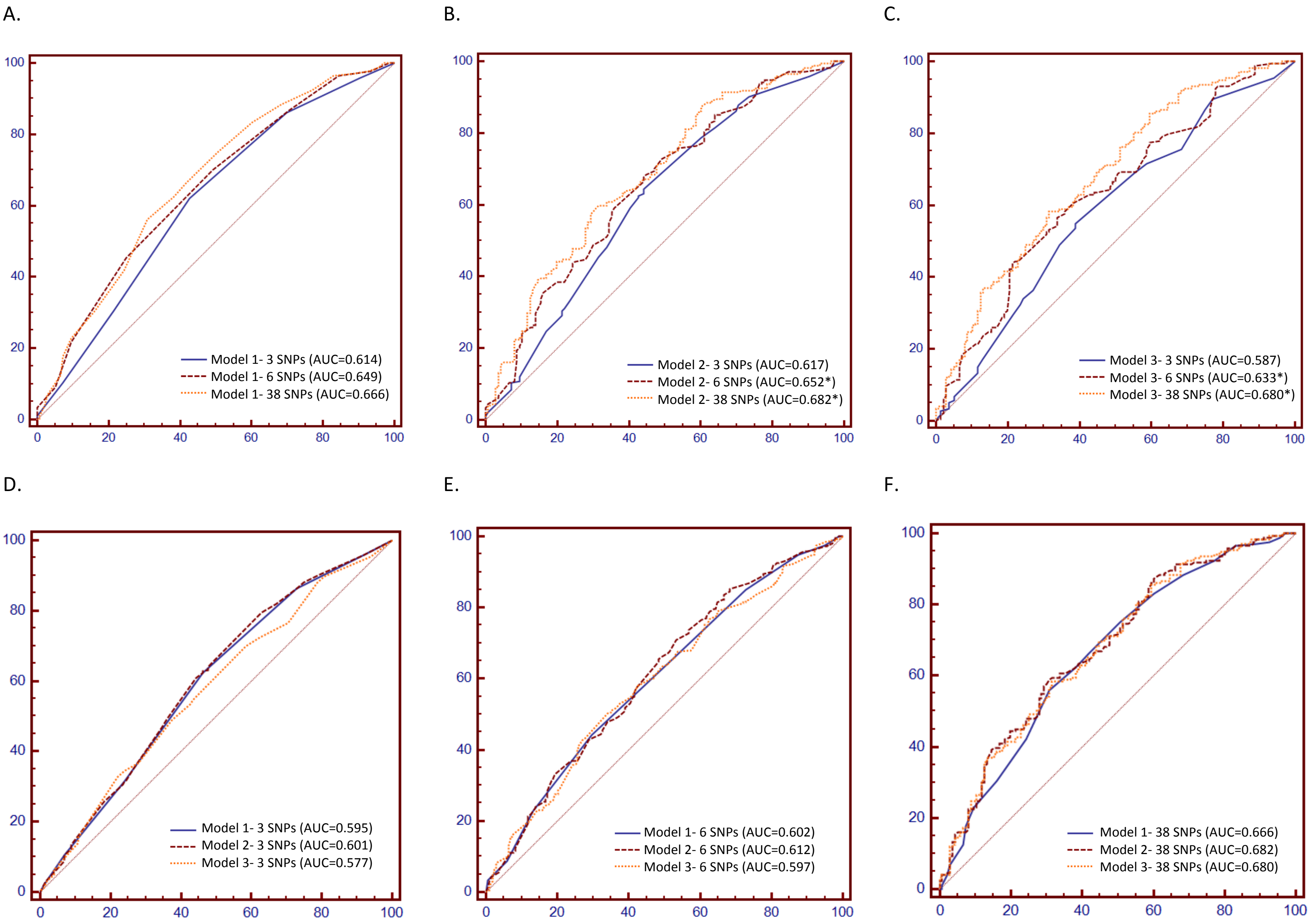

A.
Model 1- 3 SNPs (AUC=0.614)
Model 1- 6 SNPs (AUC=0.649)
Model 1- 38 SNPs (AUC=0.666)
B.
Model 2- 3 SNPs (AUC=0.617)
Model 2- 6 SNPs (AUC=0.652*)
Model 2- 38 SNPs (AUC=0.682*)
C.
Model 3- 3 SNPs (AUC=0.587)
Model 3- 6 SNPs (AUC=0.633*)
Model 3- 38 SNPs (AUC=0.680*)
D.
Model 1- 3 SNPs (AUC=0.595)
Model 2- 3 SNPs (AUC=0.601)
Model 3- 3 SNPs (AUC=0.577)
E.
Model 1- 6 SNPs (AUC=0.602)
Model 2- 6 SNPs (AUC=0.612)
Model 3- 6 SNPs (AUC=0.597)
F.
Model 1- 38 SNPs (AUC=0.666)
Model 2- 38 SNPs (AUC=0.682)
Model 3- 38 SNPs (AUC=0.680)

Figure S1. Genetic risk models 1-3 for 3, 6 and 38 SNPs. (a) Genetic risk Model 1: Count GRS (cGRS) is defined as the total sum of risk alleles present at each SNP (cGRS=∑risk alleles). (b) Genetic risk Model 2: weighted GRS (wGRS) considers both the number of risk alleles and the OR attributed to that SNP (wGRS=LN OR∑risk alleles). (c) Genetic risk Model 3: Risk relative to the average population is defined as the genotypic risk (OR^n risk alleles) divided by the average population risk (∑population frequency x genotypic risk) and calculated for each genotype combination. All models were calculated using 38 SNPs (d), 6 SNPs that reached statistical significance in stage 1 (e) and 3 SNPs significant in stage 2 (f). Population specific study ORs and allele frequencies were used to calculate GRSs. For genetic risk Models 2 and 3 (b) and (c) the 6 SNP and 38 SNP combinations significantly improved predictive power compared to 3 SNPs only. For Model 2 the difference between AUC=0.035 (95%CI 0.005-0.065, P=0.0208) and 0.065 (95%CI 0.015-0.116, P=0.0117) for 6 and 38 SNPs respectively. For Model 3, the difference between AUC=0.047 (95%CI 0.005-0.088, P=0.0282) and 0.093 (95%CI 0.027-0.159, P=0.0061) for 6 and 38 SNPs respectively. There was no significant difference within each model for any of the *n* SNP combinations (d) to (f).
