# Supplementary material for: Addressing the contribution of previously described genetic and epidemiological risk factors associated with increased prostate cancer risk and aggressive disease within men from South Africa
Source: BMC Urol. 2013 Dec 29;13:74. doi: 10.1186/1471-2490-13-74 (PMC3882498; doi:10.1186/1471-2490-13-74)
Supplement: Additional file 3: Table S2 — SAPCS study characteristics and association with prostate cancer risk. [file 1471-2490-13-74-S3.docx]

**Table S2.** SAPCS characteristics and association with prostate cancer risk.

| **Characteristic** | **Cases**  n=522 (%) | **Controls**  n=315 (%) | **Unadjusted P-value*** | **Adjusted P-value; OR (95%CI)^†^** |
| --- | --- | --- | --- | --- |
| **Population**  Tsonga  Pedi  Venda  Tswana  Ndebele  Zulu  Swati  Sotho  Xhosa | 74 (14.2)  263 (50.4)  79 (15.1)  30 (5.7)  29 (5.6)  21 (4.0)  12 (2.3)  7 (1.3)  7 (1.3) | 43 (13.7)  193 (61.3)  26 (8.3)  27 (8.6)  7 (2.2)  11 (3.5)  4 (1.3)  2 (0.6)  2 (0.6) | **0.0046** | *Reference*^‡^  0.29; 0.80 (0.52-1.21)  **0.0473**; 1.81 (1.01-3.29)  0.28; 0.70 (0.37-1.34)  0.06; 2.41 (1.01-3.29)  0.64; 1.24 (0.52-1.21)  0.26; 2.18 (0.63-10.15)  0.29; 3.28 (0.51-64.08)  0.56; 1.64 (0.34-11.83) |
| **Clinic Location**  Polokwane  SBAH/MEDUNSA  Tshilidzini | 363 (69.5)  147 (28.2)  12 (2.3) | 231 (73.3)  74 (23.5)  10 (3.2) | 0.27 | *Reference*^‡^  0.55; 1.15 (0.73-1.84)  0.05; 0.39 (0.15-1.02) |
| **Age**  Mean  Median  range  *Missing value* | 71.1  71  49-101  0 | 70.6  70  45-99  0 | 0.46 | 0.62; 1.00 (0.99-1.02) |
| **Family history PCa**  0  ≥1 first degree relative  *Missing value* | 408 (84.8)  73 (15.2)  *41* | 270 (87.9)  37 (12.1)  *8* | 0.26 | *Reference*  0.19; 1.34 (0.87-2.07) |
| **Family history any Ca**  0  ≥1 first degree relative  *Missing value* | 360 (78.6)  98 (21.4)  64 | 246 (84.8)  44 (15.2)  25 | **0.0434** | *Reference*  **0.0155**; 2.60 (1.24-5.94) |
| **Occupation**  Business  Construction (labor)  Transport  Health/Education  Hospitality  Construction (specialty)  Factory worker  Mining  Protective services  Agriculture  Unemployed  *Missing value* | 20 (4.3)  207 (44.3)  86 (18.4)  40 (8.6)  36 (7.7)  20 (4.3)  18 (3.9)  13 (2.8)  14 (3.0)  6 (1.3)  7 (1.5)  *55* | 12 (4.4)  144 (52.7)  41 (15.0)  6 (2.2)  19 (7.0)  14 (5.1)  12 (4.4)  3 (1.1)  12 (4.4)  4 (1.5)  6 (2.2)  *42* | **0.0373** | *Reference*  0.32; 0.67 (0.30-1.46)  0.93; 0.96 (0.40-2.24)  0.06; 3.05 (0.99-10.22)  0.89; 0.94 (0.36-2.41)  0.56; 0.73 (0.26-2.06)  0.78; 0.86 (0.29-2.54)  0.16; 3.31 (0.71-24.09)  0.39; 0.62 (0.20-1.84)  0.37; 0.48 (0.09-2.47)  0.39; 0.55 (0.14-2.18) |
| **Diabetes**  No  Yes  *Missing value* | 150 (65.8)  78 (34.2)  *294* | 117 (77.5)  34 (22.5)  *164* | **0.0199** | *Reference*  **0.0161**; 1.83 (1.13-3.01) |
| **Allergies**  No  Yes  *Missing value* | 202 (88.2)  27 (11.8)  *286* | 137 (90.7)  14 (9.3)  *164* | 0.54 | *Reference*  0.46; 1.32 (0.64-2.82) |
| **STD exposure**  No  Yes  *Missing value* | 295 (57.2)  221 (42.8)  *6* | 165 (52.9)  147 (47.1)  *3* | 0.26 | *Reference*  0.12; 0.79 (0.59-1.07) |
| **Traditional medicine**  No  Yes  *Missing value* | 291 (56.8)  221 (43.2)  *10* | 184 (58.8)  129 (41.2)  *2* | 0.63 | *Reference*  0.38; 1.14 (0.85-1.54) |
| **Erectile dysfunction**  No  Yes  *Missing value* | 126 (24.5)  389 (75.5)  *7* | 121 (39.2)  188 (60.8)  *6* | **<0.0001** | *Reference*  **0.0187**; 1.53 (1.07-2.18)^ |
| **Age erectile dysfunction**  Mean  Median  range  *Missing value* | 66.2  68  13-88  (218) | 66.5  68  20-87  (148) | 0.74 | 0.84; 1.00 (0.97-1.04) |
| **Age first sex**  Mean  Median  range  *Missing value* | 21.2  21  10-61  *26* | 21.2  21  12-31  *14* | 0.83 | 0.75; 1.00 (0.97-1.05) |
| **Present sex**  No  Yes  *Missing value* | 332 (64.5)  183 (35.5)  *7* | 133 (42.8)  178 (57.2)  *4* | **<0.0001** | *Reference*  **<0.0001**; 0.48 (0.34-0.68)^ |
| **Acne**  No  Yes  *Missing value* | 481 (93.4)  34 (6.6)  *7* | 298 (96.8)  10 (3.2)  *7* | 0.06 | *Reference*  0.20; 1.70 (0.78-4.03) |
| **Chest hair**  No  Yes  *Missing value* | 352 (68.5)  162 (31.5)  *8* | 221 (72.2)  85 (27.8)  *9* | 0.29 | *Reference*  0.19; 1.25 (0.90-1.74) |
| **Male Breasts**  No  Yes  *Missing value* | 278 (54.6)  231 (45.4)  *13* | 165 (54.8)  136 (45.2)  *14* | 1.0 | *Reference*  0.38; 1.15 (0.84-1.57) |
| **2D:4D Digit ratio**  2D=4D  2D>4D  4D>2D  *Missing value* | 40 (9.4)  5 (1.2)  386 (90.4)  *91* | 16 (6.0)  2 (0.7)  249 (93.3)  *48* | 0.25 | *Reference*  0.40; 0.41 (0.04-3.75)  0.11; 0.59 (0.31-1.10) |
| **Balding pattern**  No balding  Frontal  vertex  frontal + vertex  *Missing value* | 212 (44.2)  44 (9.2)  39 (8.1)  185 (38.5)  *42* | 155 (52.0)  41 (13.8)  22 (7.4)  80 (26.8)  *17* | **0.0038** | *Reference*  0.17; 0.71 (0.43-1.16)  0.68; 1.13 (0.63-2.05)  **0.0087**; 1.60 (1.13-2.28) |
| **Balding age**  ≥80  70-79  60-69  50-59  40-49  30-39  20-29  *Missing value* | 4 (1.4)  42 (15.1)  103 (36.9)  81 (29.0)  32 (11.5)  12 (4.3)  5 (1.8)  *201* | 1 (0.7)  16 (11.0)  55 (37.7)  54 (37.0)  12 (8.2)  6 (4.1)  2 (1.4)  *152* | 0.63 | *Reference*  0.84; 0.78 (0.04-7.05)  0.67; 0.60 (0.03-5.26)  0.53; 0.47 (0.02-4.22)  0.91; 0.87 (0.04-8.37)  0.76; 0.67 (0.03-7.61)  0.78; 0.67 (0.02-11.90) |
| **Red meat consumption**  No  Yes  *Missing value* | 50 (9.6)  469 (90.4)  *3* | 25 (8.0)  288 (92)  *2* | 0.50 | *Reference*  0.39; 0.80 (0.46-1.33) |
| **Aspirin usage**  No  Yes  *Missing value* | 333 (64.9)  180 (35.1)  *9* | 241 (77.0)  72 (23.0)  *2* | **0.0003** | *Reference*  **0.0026**; 1.68 (1.20-2.37) |
| **PSA**  <4 µg/L  ≥4<10 µg/L  ≥10<20 µg/L  ≥20<100 µg/L  ≥100 µg/L  *Missing value* | 9 (1.9)  22 (4.5)  50 (10.3)  162 (33.4)  242 (49.9)  *37* | 74 (24.5)  90 (29.8)  81 (26.8)  47 (15.6)  10 (3.3)  *13* | **<0.0001** | *Reference*  0.06; 2.40 (0.99-6.45)  **0.0001**; 5.51 (2.44-14.22)  **<0.0001**; 36.71 (16.50-94.22)  **<0.0001**; 228.79 (89.04-681.99) |

* Unadjusted P-value generated using Pearson's Chi-squared test with Yates' continuity correction for dichotomous variables or Welch two sample T-test for continuous variables reflects over-all difference in distribution.

^†^ P-values, odds ratios (OR) and 95% confidence intervals (CI) adjusted for age, population and a family history of prostate cancer were calculated for individual variable groups referenced against a specified (lowest risk) group using multiple logistic regression analysis.

^‡^ Reference group for multiple variable outcomes selected based on most equal distribution in cases and controls.

^§^ Reason for attending clinic is reported for all study samples present in the SAPCC database as of July 2012, which includes 592 case samples and 328 controls. Reports are received for cases and controls per study population, not per individual study sample. Hence only unadjusted analysis can be performed.

^¶^ Urological complaints includes: retention/LUTS, dysuria, obstructed flow, Haematuria, Nocturia, incontinence, increased frequency and Urinary tract infection.
