# Supplementary material for: Addressing the contribution of previously described genetic and epidemiological risk factors associated with increased prostate cancer risk and aggressive disease within men from South Africa
Source: BMC Urol. 2013 Dec 29;13:74. doi: 10.1186/1471-2490-13-74 (PMC3882498; doi:10.1186/1471-2490-13-74)
Supplement: Additional file 4: Table S3 — Case-only analysis for association between epidemiological measures and Gleason score. [file 1471-2490-13-74-S4.docx]

**Table S3.** Case-only analysis for association between epidemiological measures and Gleason score.

| **GLEASON SCORE** | **<7 (N=132)** | **=7 (N=82)** | | **>7 (N=131)** | |
| --- | --- | --- | --- | --- | --- |
|  | n (%) | n (%) | P-value; RR (95% CI)* | n (%) | P-value; RR (95% CI)* |
| **Population**  Tsonga  Pedi  venda  Tswana  Ndebele  Zulu  Swati  Sotho  Xhosa | 15 (11.4)  62 (47.0)  32 (24.2)  5 (3.8)  7 (5.3)  4 (3.0)  3 (2.3)  2 (1.5)  2 (1.5) | 8 (9.8)  42 (51.2)  9 (11.0)  2 (2.4)  7 (8.5)  8 (9.8)  2 (2.4)  1 (1.2)  3 (3.7) | *Reference*  0.49; 1.45 (0.51-4.13)  0.43; 0.61 (0.18-2.10)  0.88; 0.86 (0.13-5.78)  0.28; 2.23 (0.51-9.64)  0.06; 5.31 (0.96-29.52)  0.74; 1.41 (0.18-10.81)  0.64; 2.00 (0.11-37.95)  0.29; 4.11 (0.31-54.87) | 24 (18.3)  68 (51.9)  18 (13.7)  5 (3.8)  7 (5.3)  4 (3.1)  1 (0.8)  4 (3.1)  0 (0) | *Reference*  0.43; 0.73 (0.34-1.58)  **0.0267; 0.36 (0.15-0.89)**  0.44; 0.57 (0.14-2.38)  0.63; 0.73 (0.21-2.61)  0.74; 0.75 (0.14-4.09)  0.17; 0.19 (0.02-2.01)  0.73; 1.52 (0.14-16.51)  0.99; NaN |
| **Clinic Location**  Polokwane  SBAH/MEDUNSA  Tshilidzini | 94 (71.2)  37 (28.0)  1 (0.8) | 47 (57.3)  35 (42.7)  0 (0) | *Reference*  0.73; 0.85 (0.33-2.20)  1.00; NaN | 103 (78.6)  25 (19.1)  3 (2.3) | *Reference*  **0.0088; 0.30 (0.12-0.74)**  0.12; 6.77 (0.63-73.05) |
| **Age**  Mean  Median  range | 73.03  74  53-101 | 70.8  74  52-98 | 0.20; 0.98 (0.94-1.01) | 70.09  69  49-91 | **0.0137; 0.98 (0.94-0.99)** |
| **Family history PCa**  0  ≥1 first degree relative  *Missing value* | 98 (79.7)  25 (20.3)  *9* | 57 (82.6)  12 (17.4)  *13* | *Reference*  0.35; 0.67 (0.30-1.54) | 106 (85.5)  18 (14.5)  *7* | *Reference*  0.19; 0.63 (0.31-1.26) |
| **Family history any Ca**  0  ≥1 first degree relative  *Missing value* | 88 (72.1)  34 (27.9)  *10* | 52 (80)  13 (20)  *17* | *Reference*  0.99; NaN | 93 (76.2)  29 (23.8)  *9* | *Reference*  0.96; 1.03 (0.37-2.88) |
| **Occupation**  Health/Education  Business  Construction (labor)  Transport  Hospitality  Construction (specialty)  Factory worker  Mining  Protective services  Agriculture  Unemployed  *Missing value* | 8 (7.0)  5 (4.4)  54 (47.4)  23 (20.2)  9 (7.9)  3 (2.6)  3 (2.6)  4 (3.5)  2 (1.8)  3 (2.6)  0 (0)  *18* | 6 (7.9)  2 (2.6)  35 (46.1)  8 (10.5)  9 (11.8)  6 (7.9)  3 (3.9)  1 (1.3)  2 (2.6)  2 (2.6)  2 (2.6)  *6* | *Reference*  0.47; 0.39 (0.03-4.87)  0.74; 1.25 (0.33-4.70)  0.14; 0.30 (0.06-1.48)  0.39; 2.03 (0.41-10.14)  0.48; 2.07 (0.28-15.28)  0.56; 0.50 (0.05-4.89)  0.49; 0.41 (0.03-5.17)  0.38; 3.40 (0.22-51.96)  0.77; 1.43 (0.14-15.06)  1.00; NaN | 8 (6.8)  7 (5.9)  51 (43.2)  22 (18.6)  5 (4.2)  7 (5.9)  6 (5.1)  5 (4.2)  4 (3.4)  0 (0)  3 (2.5)  *13* | *Reference*  0.78; 1.25 (0.26-6.14)  0.83; 1.13 (0.38-3.38)  0.39; 0.58 (0.17-2.00)  0.65; 0.70 (0.15-3.27)  0.49; 1.88 (0.32-11.01)  0.71; 1.42 (0.23-8.86)  0.91; 1.10 (0.20-6.06)  0.28; 3.88 (0.34-44.62)  1.00; NaN  1.00; NaN |
| **Diabetes**  No  Yes  *Missing value* | 39 (58.2)  28 (41.8)  *65* | 21 (67.7)  10 (32.3)  *51* | *Reference*  0.49; 0.72 (0.29-1.81) | 33 (64.7)  18 (35.3)  *80* | *Reference*  0.42; 0.72 (0.33-1.59) |
| **Allergies**  No  Yes  *Missing value* | 61 (91.0)  6 (9.0)  *65* | 29 (93.5)  2 (6.5)  *51* | *Reference*  0.41; 1.18 (0.32-4.40) | 47 (90.4)  5 (9.6)  *79* | *Reference*  0.81; 1.18 (0.32-4.40) |
| **STD exposure**  No  Yes  *Missing value* | 72 (55.0)  59 (45.0)  *1* | 49 (59.8)  33 (40.2)  *0* | *Reference*  0.20; 0.66 (0.35-1.24) | 71 (54.6)  59 (45.4)  *1* | *Reference*  0.77; 0.92 (0.55-1.56) |
| **Traditional medicine**  No  Yes  *Missing value* | 69 (52.7)  62 (47.3)  *1* | 53 (66.3)  27 (33.8)  *2* | *Reference*  0.0136; 0.44 (0.23-0.84) | 73 (57.0)  55 (43.0)  *3* | *Reference*  0.32; 0.76 (0.45-1.30) |
| **Erectile dysfunction**  No  Yes  *Missing value* | 33 (25.0)  99 (75.0)  *0* | 17 (20.7)  65 (79.3)  *0* | *Reference*  0.69; 1.16 (0.55-2.46) | 31 (24.2)  97 (75.8)  *3* | *Reference*  0.77; 0.91 (0.50-1.67) |
| **Age ED**  Mean  Median  range  *Missing value* | 66.96  68  40-88  *55* | 65.58  68  13-80  *27* | **0.0322; 1.09 (1.01-1.20)** | 65.77  66  45-80  *49* | 0.14; 1.05 (0.98-1.13) |
| **Age first sex**  Mean  Median  range  *Missing value* | 21.57  22  13-61  *6* | 20.54  20  14-30  *3* | 0.33; 0.96 (0.88-1.05) | 21.23  22  10-30  *5* | 0.57; 0.98 (0.92-1.05) |
| **Present sex**  No  Yes  *Missing value* | 79 (59.8)  53 (40.2)  *0* | 59 (72.0)  23 (28.0)  *0* | *Reference*  **0.0395; 0.49 (0.25-0.97)** | 90 (69.8)  39 (30.2)  *2* | *Reference*  **0.0154; 0.50 (0.29-0.88)** |
| **Acne**  No  Yes  *Missing value* | 122 (93.1)  9 (6.9)  *1* | 78 (95.1)  4 (4.9)  *0* | *Reference*  **0.0379; 0.15 (0.02-0.90)** | 122 (93.8)  8 (6.2)  *1* | *Reference*  0.35; 0.55 (0.16-1.95) |
| **Chest hair**  No  Yes  *Missing value* | 91 (69.5)  40 (30.5)  *1* | 58 (70.7)  24 (29.3)  *0* | *Reference*  0.65; 1.18 (0.58-2.40) | 85 (64.9)  46 (35.1)  *0* | *Reference*  0.49; 1.23 (0.69-2.20) |
| **Male Breasts**  No  Yes  *Missing value* | 77 (60.2)  51 (39.8)  *4* | 47 (58.8)  33 (41.3)  *2* | *Reference*  0.09; 1.80 (0.91-3.58) | 61 (46.9)  69 (53.1)  *1* | *Reference*  **0.0176; 1.98 (1.13-3.49)** |
| **2D:4D Digit ratio**  2D=4D  2D>4D  4D>2D  *Missing value* | 11 (10.3)  0 (0)  96 (89.7)  *25* | 2 (3.0)  0 (0)  65 (97.0)  *15* | *Reference*  1.00; NaN  0.17; 3.04 (0.63-14.73) | 13 (12.1)  1 (0.9)  93 (86.9)  *24* | *Reference*  1.00; NaN  0.75; 0.86 (0.35-2.13) |
| **Balding pattern**  No balding  Frontal  vertex  frontal + vertex  *Missing value* | 46 (37.7)  7 (5.7)  14 (11.5)  55 (45.1)  *10* | 29 (37.7)  6 (7.8)  8 (10.4)  34 (44.2)  *5* | *Reference*  0.73; 1.28 (0.32-5.16)  0.70; 0.81 (0.26-2.45)  0.92; 1.04 (0.51-2.09) | 61 (49.2)  14 (11.3)  5 (4.0)  44 (35.5)  *7* | *Reference*  0.35; 1.72 (0.55-5.38)  **0.0400; 0.30 (0.10-0.95)**  0.17; 0.66 (0.37-1.20) |
| **Balding age**  ≥70  60-69  50-59  40-49  30-39  20-29  *Missing value* | 15 (18.8)  26 (32.5)  25 (31.3)  12 (15.0)  2 (2.5)  0 (0)  *52* | 9 (17.6)  18 (35.3)  11 (21.6)  7 (13.7)  3 (5.9)  3 (5.9)  *31* | *Reference*  0.74; 0.82 (0.25-2.67)  0.32; 0.50 (0.13-1.96)  0.10; 0.25 (0.05-1.30)  0.91; 0.88 (0.09-8.24)  1.00; NaN | 4 (6.9)  23 (39.7)  20 (34.5)  7 (12.1)  2 (3.4)  2 (3.4)  *73* | *Reference*  0.33; 1.91 (0.52-7.08)  0.50; 1.61 (0.40-6.59)  0.75; 1.28 (0.27-6.07)  0.71; 1.59 (0.14-18.70)  1.00; NaN |
| **Red meat consumption**  No  Yes  *Missing value* | 12 (9.1)  120 (90.9)  *0* | 7 (8.5)  75 (91.5)  *0* | *Reference*  0.58; 0.75 (0.26-2.10) | 13 (10.0)  117 (90.0)  *1* | *Reference*  0.40; 0.69 (0.29-1.65) |
| **Aspirin usage**  No  Yes  *Missing value* | 94 (71.8)  37 (28.2)  *1* | 47 (59.5)  32 (40.5)  *3* | *Reference*  0.25; 1.50 (0.76-2.99) | 83 (63.8)  47 (36.2)  *1* | *Reference*  0.15; 1.55 (0.86-2.79) |
| **PSA**  <10 µg/L  ≥10<20 µg/L  ≥20<100 µg/L  ≥100 µg/L  *Missing value* | 9 (7.4)  17 (14.0)  48 (39.7)  47 (38.8)  *11* | 4 (5.0)  8 (10.0)  27 (33.8)  41 (51.3)  *2* | *Reference*  0.73; 0.73 (0.12-4.45)  0.63; 1.44 (0.33-6.27)  0.19; 2.64 (0.61-11.32) | 5 (4.0)  8 (6.5)  39 (31.5)  72 (58.1)  *7* | *Reference*  0.81; 0.83 (0.18-3.74)  0.35; 1.81 (0.52-6.36)  0.07; 3.19 (0.91-11.16) |

* P-value, relative risk (RR) and 95% confidence intervals (CI) estimated using mutinomial logistic regression adjusted for age, population and family history of prostate cancer. “Present Sex” also adjusted for erectile dysfunction.
