# Supplementary material for: Addressing the contribution of previously described genetic and epidemiological risk factors associated with increased prostate cancer risk and aggressive disease within men from South Africa
Source: BMC Urol. 2013 Dec 29;13:74. doi: 10.1186/1471-2490-13-74 (PMC3882498; doi:10.1186/1471-2490-13-74)
Supplement: Additional file 5: Table S4 — Case-only analysis for association between epidemiological measures and tumor grade. [file 1471-2490-13-74-S5.docx]

**Table S4.** Case-only analysis for association between epidemiological measures and tumor grade.

| **TUMOR GRADE*** | **Well (N=71)** | **Moderate (N=155)** | | **Poor (N=79)** | |
| --- | --- | --- | --- | --- | --- |
|  | n (%) | n (%) | P-value; RR (95% CI)^†^ | n (%) | P-value; RR (95% CI)^†^ |
| **Population**  Tsonga  Pedi  Venda  Tswana  Ndebele  Zulu  Swati  Sotho  Xhosa | 9 (12.7)  44 (62.0)  6 (8.5)  2 (2.8)  5 (7.0)  4 (5.6)  0 (0)  0 (0)  1 (1.4) | 23 (14.8)  74 (47.7)  26 (16.8)  6 (3.9)  10 (6.5)  8 (5.2)  4 (2.6)  2 (1.3)  2 (1.3) | *Reference*  0.58; 0.77 (0.31-1.93)  0.29; 1.99 (0.56-7.08)  0.81; 1.25 (0.20-7.79)  0.98; 0.98 (0.23-4.12)  0.87; 1.17 (0.19-7.26)  1.00; NaN  1.00; NaN  1.00; NaN | 12 (15.2)  43 (54.4)  20 (25.3)  1 (1.3)  1 (1.3)  0 (0)  2 (2.5)  0 (0)  0 (0) | *Reference*  0.54; 0.73 (0.27-2.00)  0.14; 2.73 (0.72-10.31)  0.39; 3.22 (0.02-4.24)  0.15; 0.17 (0.02-1.86)  1.00; NaN  1.00; NaN  1.00; NaN  1.00; NaN |
| **Clinic Location**  Polokwane  SBAH/MEDUNSA  Tshilidzini | 46 (64.8)  24 (33.8)  1 (1.4) | 109 (70.3)  44 (28.4)  2 (1.3) | *Reference*  0.69; 1.22 (0.46-3.25)  0.58; 0.49 (0.04-6.35) | 71 (89.9)  3 (3.8)  5 (6.3) | *Reference*  0.07; 0.25 (0.06-1.11)  0.72; 1.53 (0.15-15.96) |
| **Age**  Mean  Median  range | 71.5  72  54-101 | 71.4  71  52-98 | 0.93; 1.00 (0.96-1.04) | 71.2  71  53-89 | 0.52; 0.99 (0.94-1.03) |
| **Family history PCa**  0  ≥1 first degree relative  *Missing value* | 51 (89.5)  6 (10.5)  *14* | 113 (75.8)  36 (24.2)  *6* | *Reference*  **0.0260; 2.90 (1.14-7.43)** | 62 (83.8)  12 (16.2)  *5* | *Reference*  0.29; 1.79 (0.61-5.22) |
| **Family history any Ca**  0  ≥1 first degree relative  *Missing value* | 46 (82.1)  10 (17.9)  *15* | 97 (67.4)  47 (32.6)  *11* | *Reference*  0.48; 1.56 (0.46-5.34) | 57 (77.0)  17 (23.0)  *5* | *Reference*  0.85; 0.87 (1.20-3.76) |
| **Occupation**  Health/Education  Business  Construction (labor)  Transport  Hospitality  Construction (specialty)  Factory worker  Mining  Protective services  Agriculture  Unemployed  *Missing value* | 4 (6.3)  3 (4.8)  25 (39.7)  16 (25.4)  4 (6.3)  2 (3.2)  2 (3.2)  1 (1.6)  6 (9.5)  0 (0)  0 (0)  *8* | 10 (7.1)  2 (1.4)  61 (43.3)  22 (15.3)  20 (14.2)  6 (4.3)  8 (5.7)  3 (2.3)  5 (3.5)  3 (2.1)  1 (0.7)  *14* | *Reference*  0.14; 0.13 (0.01-1.88)  0.57; 1.47 (0.39-5.52)  0.82; 0.85 (0.20-3.61)  0.34; 2.24 (0.43-11.65)  0.90; 1.14 (0.14-9.69)  0.75; 1.43 (0.17-12.29)  0.91; 1.17 (0.08-17.21)  0.45; 0.51 (0.09-2.99)  1.00; NaN  1.00; NaN | 7 (10.1)  1 (1.4)  43 (62.3)  8 (11.6)  1 (1.4)  1 (1.4)  3 (4.3)  2 (2.9)  0 (0)  0 (0)  3 (4.3)  *10* | *Reference*  0.25; 0.21 (0.01-3.03)  0.78; 1.22 (0.30-4.90)  0.44; 0.53 (0.11-2.62)  0.17; 0.17 (0.01-2.14)  0.57; 0.44 (0.03-7.30)  0.69; 1.61 (0.15-17.05)  0.93; 1.13 (0.07-18.51)  1.00; NaN  1.00; NaN  1.00; NaN |
| **Diabetes**  No  Yes  *Missing value* | 15 (57.7)  11 (42.3)  *45* | 43 (62.3)  26 (37.7)  *86* | *Reference*  0.53; 0.73 (0.28-1.94) | 23 (71.9)  9 (28.1)  *47* | *Reference*  0.21; 0.48 (0.15-1.50) |
| **Allergies**  No  Yes  *Missing value* | 25 (96.2)  1 (3.8)  *45* | 60 (85.7)  10 (14.3)  *85* | *Reference*  0.99; NaN | 29 (90.6)  3 (9.4)  *47* | *Reference*  0.99; NaN |
| **STD exposure**  No  Yes  *Missing value* | 39 (55.7)  31 (44.3)  *1* | 88 (56.8)  67 (43.2)  *0* | *Reference*  0.90; 1.04 (0.55-1.98) | 48 (61.5)  30 (38.5)  *1* | *Reference*  0.58; 0.81 (0.81-0.39) |
| **Traditional medicine**  No  Yes  *Missing value* | 36 (51.4)  34 (48.6)  *1* | 88 (58.3)  63 (41.7)  *4* | *Reference*  0.30; 0.71 (0.37-1.35) | 41 (52.6)  37 (47.4)  *1* | *Reference*  0.86; 0.94 (0.46-1.92) |
| **Erectile dysfunction**  No  Yes  *Missing value* | 14 (19.7)  57 (80.3)  *0* | 45 (29.2)  109 (70.8)  *1* | *Reference*  0.10; 0.52 (0.24-1.14) | 21 (27.3)  56 (72.7)  *2* | *Reference*  0.19; 0.56 (0.23-1.34) |
| **Age ED**  Mean  Median  range  *Missing value* | 64.7  67  13-88  *24* | 67.1  68  20-86  *66* | **0.0122; 1.13 (1.03-1.25)** | 67.5  69  52-80  *34* | **0.0477; 1.13 (1.00-1.26)** |
| **Age first sex**  Mean  Median  range  *Missing value* | 21.7  22  13-61  *4* | 21.6  22  14-32  *6* | 0.89; 0.99 (0.92-1.07) | 21.6  22  16-30  *7* | 0.84; 0.99 (0.89-1.10) |
| **Present sex**  No  Yes  *Missing value* | 40 (57.1)  30 (42.9)  *1* | 92 (59.7)  62 (40.3)  *1* | *Reference*  0.52; 0.81 (0.43-1.54) | 45 (57.7)  33 (42.3)  *1* | *Reference*  0.77; 0.90 (0.43-1.85) |
| **Acne**  No  Yes  *Missing value* | 65 (91.5)  6 (8.5)  *0* | 148 (96.1)  6 (3.9)  *1* | *Reference*  0.94; 0.94 (0.15-5.82) | 77 (98.7)  1 (1.3)  *1* | *Reference*  0.98; 1.04 (0.07-14.97) |
| **Chest hair**  No  Yes  *Missing value* | 54 (76.1)  17 (23.9)  *0* | 105 (69.1)  47 (30.9)  *3* | *Reference*  0.63; 1.20 (0.57-2.52) | 45 (57.0)  34 (43.0)  *0* | *Reference*  0.42; 1.40 (0.62-3.13) |
| **Male Breasts**  No  Yes  *Missing value* | 33 (48.5)  35 (51.5)  *3* | 81 (52.9)  72 (47.1)  *2* | *Reference*  0.16; 0.61 (0.30-1.22) | 29 (37.2)  49 (62.8)  *1* | *Reference*  0.65; 0.84 (0.38-1.82) |
| **2D:4D Digit ratio**  2D=4D  2D>4D  4D>2D  *Missing value* | 5 (8.3)  0 (0)  55 (91.7)  *11* | 11 (8.5)  0 (0)  119 (91.5)  *25* | *Reference*  1.00; NaN  0.96; 1.03 (0.32-3.34) | 7 (10.6)  1 (1.5)  58 (87.9)  *13* | *Reference*  1.00; NaN  0.63; 0.73 (0.21-2.59) |
| **Balding pattern**  No balding  Frontal  vertex  frontal + vertex  *Missing value* | 30 (43.5)  4 (5.8)  6 (8.7)  29 (42.0)  *2* | 55 (38.7)  7 (4.9)  15 (10.6)  65 (45.8)  *13* | *Reference*  0.71; 1.33 (0.30-5.96)  0.55; 1.45 (0.44-4.80)  0.34; 1.41 (0.69-2.88) | 36 (50.0)  8 (11.1)  5 (6.9)  23 (31.9)  *7* | *Reference*  0.42; 1.82 (0.42-7.91)  0.60; 0.68 (0.16-2.89)  0.20; 0.58 (0.26-1.33) |
| **Balding age**  ≥70  60-69  50-59  40-49  30-39  20-29  *Missing value* | 4 (11.4)  14 (40.0)  6 (17.1)  7 (20.0)  4 (11.4)  0 (0)  *36* | 19 (19.8)  39 (40.6)  28 (29.2)  7 (7.3)  2 (2.1)  1 (1.0)  *59* | *Reference*  0.66; 0.75 (0.20-2.73)  0.76; 1.27 (0.27-6.06)  0.51; 0.55 (0.09-3.30)  **0.0477; 0.12 (0.01-0.98)**  1.00; NaN | 4 (11.1)  18 (50.0)  9 (25.0)  4 (11.1)  0 (0)  1 (2.8)  *43* | *Reference*  0.86; 1.16 (0.23-5.80)  0.85; 1.20 (0.18-8.18)  0.98; 0.98 (0.12-8.15)  0.99; NaN  1.00; NaN |
| **Red meat consumption**  No  Yes  *Missing value* | 6 (8.5)  65 (91.5)  *0* | 9 (5.8)  145 (94.2)  *1* | *Reference*  0.20; 2.11 (0.67-6.69) | 7 (8.9)  72 (9.1)  *0* | *Reference*  0.51; 1.50 (0.45-4.99) |
| **Aspirin usage**  No  Yes  *Missing value* | 46 (66.7)  23 (29.9)  *2* | 91 (59.9)  61 (40.1)  *3* | *Reference*  0.98; (1.85-0.89) | 58 (73.4)  21 (26.6)  *0* | *Reference*  0.80; 1.11 (0.48-2.58) |
| **PSA**  <10 µg/L  ≥10<20 µg/L  ≥20<100 µg/L  ≥100 µg/L  *Missing value* | 9 (13.4)  7 (10.4)  23 (34.3)  28 (41.8)  *4* | 8 (5.5)  16 (11.0)  55 (37.7)  67 (45.9)  *9* | *Reference*  0.13; 3.29 (0.69-15.70)  0.23; 2.08 (0.63-6.81)  0.10; 2.69 (0.83-8.73) | 2 (2.6)  5 (6.5)  23 (29.9)  47 (61.0)  *2* | *Reference*  0.48; 2.23 (0.24-20.42)  0.18; 3.22 (0.58-17.74)  **0.0246; 6.84 (1.28-36.56)** |

* Tumor grade based on histological reports of tumor differentiation status.

^†^ P-value, relative risk (RR) and 95% confidence intervals (CI) estimated using mutinomial logistic regression adjusted for age, family history of prostate cancer and population. “Present Sex” also adjusted for erectile dysfunction.
